# Production and Characterization of Nanocellulose from Maguey (*Agave cantala*) Fiber

**DOI:** 10.3390/polym16101312

**Published:** 2024-05-07

**Authors:** Erwin C. Sumarago, Mary Frahnchezka M. dela Cerna, Andrea Kaylie B. Leyson, Noel Peter B. Tan, Kendra Felizimarie Magsico

**Affiliations:** 1 Department of Chemical Engineering, University of San Carlos, Cebu City 6000, Philippines; erwincsumarago@gmail.com (E.C.S.); mfrahnz@gmail.com (M.F.M.d.C.); andrealeyson@gmail.com (A.K.B.L.); 2 Center for Advanced New Materials, Engineering, and Emerging Technologies (CANMEET), University of San Agustin, Iloilo City 5000, Philippines; kfmagsico@usa.edu.ph

**Keywords:** *Agave cantala*, maguey fiber, alkali treatment, bleaching, acid hydrolysis, nanocellulose, nanocrystal

## Abstract

Plant fibers have been studied as sources of nanocellulose due to their sustainable features. This study investigated the effects of acid hydrolysis parameters, reaction temperature, and acid concentration on nanocellulose yield from maguey (*Agave cantala*) fiber. Nanocellulose was produced from the fibers via the removal of non-cellulosic components through alkali treatment and bleaching, followed by strong acid hydrolysis for 45 min using sulfuric acid (H_2_SO_4_). The temperature during acid hydrolysis was 30, 40, 50, and 60 °C, and the H_2_SO_4_ concentration was 40, 50, and 60 wt. % H_2_SO_4_. Results showed that 53.56% of raw maguey fibers were isolated as cellulose, that is, 89.45% was α-cellulose. The highest nanocellulose yield of 81.58 ± 0.36% was achieved from acid hydrolysis at 50 °C using 50 wt. % H_2_SO_4_, producing nanocellulose measuring 8–75 nm in diameter and 72–866 nm in length, as confirmed via field emission scanning electron microscopy (FESEM) and transmission electron microscopy (TEM) analysis. Fourier-transform infrared spectroscopy (FTIR) analysis indicated the chemical transformation of fibers throughout the nanocellulose production process. The zeta potential analysis showed that the nanocellulose had excellent colloidal stability with a highly negative surface charge of −37.3 mV. Meanwhile, X-ray diffraction (XRD) analysis validated the crystallinity of nanocellulose with a crystallinity index of 74.80%. Lastly, thermogravimetric analysis (TGA) demonstrated that the inflection point attributed to the cellulose degradation of the produced nanocellulose is 311.41 °C.

## 1. Introduction

Nanocellulose is one of today’s most prominent sustainable materials due to its numerous appealing properties, such as biodegradability, high mechanical capabilities, excellent biocompatibility, and many more [1]. It refers to a natural cellulose nanomaterial extracted from plant cell walls with nano-scale dimensions of less than 100 nanometers in diameter. This nanomaterial offers the potential for surface modification due to its abundant and highly reactive hydroxyl groups, providing an opportunity to introduce specific chemical functionalities [2]. It can be classified into three main categories: nanocrystalline cellulose, nanofibrillated cellulose, and bacterial cellulose [3]. Nanocellulose properties include high surface area, excellent strength, good stiffness, and sustainable features such as eco-friendliness, biocompatibility, non-toxicity, and renewable characteristics [4]. With its sustainable nature and excellent mechanical properties, its application ranges from material science to biomedical engineering [5]. One primary application of nanocellulose is as a polymer matrix reinforcement in biocomposite processing due to its crystal size and versatility in chemical surface modifications [6]. Nanocellulose crystals are used as reinforcing agents in various polymer matrices like polyurethane and starch, forming mechanically durable polymer nanocomposites [7].

The primary source for industrial cellulose production is wood pulp from hardwoods and softwoods [8]. However, since it takes several years for trees to become a source of cellulose, annual plants are used as an alternative source for cellulose instead of wood [9]. The search for alternative sources of cellulose fibers should be directed to plants with large biomass in which the production is low-cost, the usage time is shorter, and the source material is renewable and biodegradable [9]. Plant fibers qualify these characteristics, making them extensively studied as an alternative source for nanocellulose.

Cellulose, the essential structural component in plant fiber, is isolated through an alkali protocol, which involves alkali treatment followed by bleaching [10]. Alkali treatment is the process of cellulose isolation that utilizes sodium hydroxide solution to treat the plant fibers, removing the hemicellulose, lignin, extractive substances, and waxes and oil covering the cell walls of plant fibers [11]. This is followed by a bleaching process using oxidizing chemicals like hypochlorite and peroxide that removes the colored lignin and decomposed wax materials from the cellulosic residues of the plant fibers [12].

Nanocellulose can be produced from cellulose via various methods based on the material source and final intended application. These include chemical methods like acid hydrolysis, enzymatic hydrolysis, subcritical water hydrolysis, and 2,2,6,6-tetramethylpiperidine 1-oxyl (TEMPO) oxidation, and mechanical methods like grinding, cryo crushing, and steam explosion [1,13]. Acid hydrolysis, the most common and effective method of nanocellulose production, involves using diluted or concentrated acids to hydrolyze the amorphous regions of the cellulose [14]. Hydrolysis techniques tend to remove the amorphous regions in the fiber, producing nanocellulose with higher crystallinity [15]. Studies have reported that nanocellulose produced via acid hydrolysis has a higher crystallinity index and is of smaller sizes compared with nanocellulose produced using other methods [16]

Acid hydrolysis is carried out primarily by using concentrated acids to avoid the need for high pressure and temperature to hydrolyze the amorphous regions of cellulose effectively. Employing concentrated acids like sulfuric, sulfurous, hydrochloric, hydrofluoric, phosphoric, nitric, and formic acid facilitates the generation of high yields of nanocrystalline cellulose with enhanced adaptability to different raw materials, as opposed to the utilization of diluted acids [17]. The most preferred acid is sulfuric acid (H_2_SO_4_) because the esterification of the hydroxyl group by sulfate ions causes the cellulose nanocrystals to be strongly isolated. However, the production method using concentrated acids presents significant drawbacks owing to the corrosive and toxic nature of acids involved together with the generation of acid wastewater, which necessitates the implementation of acid waste recycling to mitigate the cost associated with acid waste disposal [1].

Numerous studies have reported the pivotal role of hydrolysis parameters, specifically acid concentration, temperature, and hydrolysis duration, in influencing the yield of nanocellulose [14]. According to the study by Bacha (2022) on the effects of acid hydrolysis process parameters for extracting nanocellulose from teff straw, the reported findings reveal that under constant reaction times and temperature conditions, the yield of nanocellulose exhibits a decreasing trend with increasing acid concentration [18]. This phenomenon implies an over-degradation of the cellulose due to heightened acid concentrations leading to the decomposition of the crystalline regions within the cellulose. Furthermore, the study also reported the effects of increasing temperature in terms of this facilitating acid–fiber interaction, where higher temperature aids deeper acid penetration into the fiber structure and subsequently degrades its components. Moreover, it was reported that, under constant temperature and acid concentrations, a prolonged hydrolysis duration results in a marginal decrease in the yield. This can be attributed to the extended contact time between the cellulose and acid, which facilitates deeper acid diffusion into the cellulose components, subsequently degrading the crystalline regions, reducing the yield [18].

The effects of varying the hydrolysis parameters as independent variables on the nanocellulose yield and the corresponding optimized conditions depend on factors such as the type, composition, and purity of raw material used, the optimization method implemented, and the range of the conditions being investigated [14]. Consequently, it is imperative to determine the best conditions resulting in high nanocellulose yields for the acid hydrolysis of a new and different raw material, which is, in this case, the maguey (*Agave cantala*) fiber.

*Agave cantala*, locally known as, “maguey”, is a plant abundant in fiber belonging to the *Agavaceae* family, most likely native to Mexico. *Agave cantala* is an *Agave* species that has been cultivated in the Philippines. Maguey plant fibers have exemplary properties such as length, strength, and luster and are characterized by their low density, high tenacity, and high moisture absorbency compared with other leaf fibers [19,20]. In the Philippines, these fibers were used to manufacture twines and ropes for fishing and agriculture. Unfortunately, these products are not widely sold anymore [21]. There are minimal studies conducted on the usage of *Agave* species as a raw material for nanocellulose production, such as *Agave tequilana* weber bagasse by Palacios Hinestroza, *Agave americana* leaves by Ortega et al., and *Agave angustifolia* leaves by Rosli et al. [7,22,23]. However, as of the time of writing, there has yet to be an existing study on the potential of using the maguey plant as a source of nanocellulose. Moreover, there is no study on the effects of hydrolysis parameters, sulfuric acid concentration, and temperature, on the yield of nanocellulose derived from this *Agave* species. Thus, this research aims to fill the knowledge gap on the production of nanocellulose from the maguey plant, which is essential to future research on exploiting local non-woody renewable sources of nanocellulose like the maguey fiber.

This study investigated the potential of using maguey fibers as an alternative source of nanocellulose through a series of processes, starting with the preparation of a raw maguey fiber sample, removal of non-cellulosic components of the fiber, and strong acid hydrolysis of cellulose from the fibers. The objective of this research is to investigate the effects of acid hydrolysis parameters, sulfuric acid concentration (40, 50, 60 wt. % H_2_SO_4_), and reaction temperature (30, 40, 50, and 60 °C) on the nanocellulose yield at a constant reaction time of 45 min. Further investigations were carried out on the chemical and physical properties of the nanocellulose obtained from the hydrolysis conditions that gave the highest yield.

## 2. Materials and Methods

Maguey fiber samples (MFS) were obtained via natural retting from the *Agave cantala* plant species, locally known as “maguey” in Tabogon, Cebu, Philippines. The collection and utilization of such plant species adhered to the local, national, and international guidelines and regulations. The chemical reagents used included sodium hydroxide pellets (98% NaOH, HIMEDIA GRM 467, Nashik, India), sodium hypochlorite (7% NaClO stock solution, Parañaque City, Philippines), sulfuric acid (98% H_2_SO_4_, AJAX 534, Scoresby, Australia), methanol (100% CH_3_OH, Scharlau ExpertQ^®^, Quezon City, Philippines), and acetic acid (CH_3_COOH, glacial, AJAX 01, Scoresby, Australia). These chemicals were used without further purification.

### 2.1. Drying and Size Reduction for Maguey Fibers

The researchers manually harvested the maguey leaves. The leaves were then retted via natural water retting to obtain their fibers. The obtained raw maguey fibers were washed three times using distilled water to remove remaining impurities before actual experimental execution and then dried in direct sunlight for 8 h. The dried maguey fibers were cut into sections measuring 3–5 cm long and then oven-dried at 80 °C or until a moisture content of <10% was achieved. A moisture content of 5.34% was achieved, as shown in Section S.I.1 in the Supplementary Material. The maguey fibers were then cut into 2–2.5 mm length pieces as part of the preparation before being pulverized in Thomas Model 4 Wiley^®^ Mill, Thomas Scientific, Swedesboro, NJ, USA, with a 1 mm screen plate. Maguey fibers were then sieved for 5 min using an ISO 3310-1 Laboratory Test Sieve, Endecotts Ltd., London, UK, sieve shaker with sieve sizes of 850, 450, 250, and 180 µm. The obtained mean particle size diameter was 582.6820 µm, as shown in Table S.I.2 in the Supplementary Material.

### 2.2. Removal of Non-Cellulosic Components of Maguey Fibers

#### 2.2.1. Alkali Treatment

A series of alkali treatments with sodium hydroxide (4% *w*/*v* NaOH) were performed on MFS. In total, 100 g of MFS was treated with 2500 mL of NaOH solution (1:25 g/mL ratio) at 75 °C for 2 h in a laboratory water bath (Model No. WNB 7, Memmert GmbH, Schwabach, Germany) with intermittent stirring every 15 min [7]. After heating, the mixture was quenched in iced water until 30 °C. The cooled samples were then filtered using cheesecloth and washed with distilled water until a filtrate of pH 7 was achieved. The wet alkali-treated MFS was retreated under the same process and conditions for the second alkali treatment. With pH 7, the alkali-treated MFS was oven-dried at 105 °C for 3 h and then further dried at 80 °C for another 3 h.

#### 2.2.2. Bleaching

The dried alkali-treated MFS with an approximate mass of 78.37 g was treated with 783.7 mL of 5% aqueous NaClO solution at 70 ℃ to room temperature for 1 h (1:10 g/mL ratio) with intermittent stirring every 15 min. The process was carried out at room temperature under a fume hood. After an hour of bleaching, the bleached MFS was filtered using cheesecloth and thoroughly washed with distilled water until a filtrate of pH 7 was reached. The filtered bleached MFS was oven-dried at 80 ℃ for 3 h. The mass of dried bleached MFS ($m_{C}$) is the amount of cellulose obtained. From the obtained cellulose, the MFS cellulose yield ($Y_{C}$) was calculated using Equation (1):

(1) $$\%\;Cellulose\;yield\;(Y_{C})=\frac{m_{C}}{m_{MFS}}\times 100\%$$

where *Y_C_* is the cellulose yield from MFS, *m_C_* is the mass of cellulose (g), and *m_MFS_* is the mass of MFS (g).

### 2.3. Determination of the Chemical Composition of Cellulose

#### 2.3.1. Determination of Holocellulose Content

Holocellulose refers to the total alpha and hemicelluloses in the extracted cellulose [24]. The holocellulose of the cellulose from MFS was extracted after 3 h of water bath shaking [25]. The process was explicitly carried out by weighing ~1 g of the cellulose sample and mixing with 40 mL of distilled water. To the mixture, 5 g of 0.1269 mM NaClO and 0.25 mL of glacial acetic acid were added, and placed it in a water bath shaker (Model No. SHZ-88 Laboratory Thermostatic Shaking Water Bath, Ningbo, China) for 1 h at 70 °C. The process of adding NaClO and CH_3_COOH was repeated every hour for 3 h, resulting in a total of 15 g of 0.1269 mM NaClO and 0.75 mL of glacial acetic acid in a 40 mL mixture of distilled water and a gram of cellulose [25].

The mixture was then cooled in the chiller to nearly 5 °C for at least 1 h. After cooling, the mixture was then brought to room temperature and then washed with a water–methanol mixture (1:1 *v*/*v* ratio) over Whatman No. 42, Merck KGaA, Darmstadt, Germany filter paper until a filtrate of pH 7 was obtained. The residue in the pre-weighed filter paper was then oven-dried at 40 °C until a constant weight was achieved. The mass obtained from the residue is the amount of holocellulose from the cellulose. Holocellulose content was calculated using Equation (2):

(2) $$\%\;Holocellulose\;content\;(\%\;HC)=\frac{m_{HC}}{m_{cellulose}}\times 100\;\%$$

where % HC is the holocellulose content of cellulose from MFS, *m_HC_* is the mass of oven-dried holocellulose (g), and *m_cellulose_* is the mass of cellulose sample (g).

#### 2.3.2. Determination of α-Cellulose Content

α-cellulose content of the produced cellulose was determined by placing the previously dried holocellulose in a mortar and adding 7 mL of 17.5 wt. % NaOH solution [25]. The contents in the mortar were then mashed slowly for 8 min in a circular motion. Afterward, another 7 mL of 17.5 wt. % NaOH solution was added to the mixture and mashed for another 8 min. The mashed sample in the mortar was transferred to a 100 mL beaker. A total of 40 mL of distilled water was added to the sample in the beaker and mixed thoroughly for 5 min. The mixture was then filtered over Whatman No. 42 filter paper and washed with a mixture of distilled water and 10% acetic acid until the residue became off-white. The residue was oven-dried at 40 ℃ until a constant weight was achieved. The mass obtained after drying is the mass of the dried α-cellulose (*m_C_*). The α-cellulose content was calculated using Equation (3):

(3) $$\%\;\alpha -cellulose\;content\;(\%\;C)=\frac{m_{C}}{m_{cellulose}}\times 100\;\%$$

where % C is the α-cellulose content of cellulose from MFS, *m_C_* is mass of the dried α-cellulose (g), and *m_cellulose_* is the mass of the cellulose sample (g).

#### 2.3.3. Determination of Hemicellulose Content

The amount of hemicellulose is the difference between holocellulose and α-cellulose amounts. Using Equation (4), the hemicellulose content (% H) was determined:

(4) $$\%\;Hemicellulose\;\left(\%\;H\right)=\%\;HC-\%\;C$$

#### 2.3.4. Determination of Lignin and Other Extractive Contents

Having determined the hemicellulose and α-cellulose content, by difference, the amount of lignin together with the other extractives was also determined. The content lignin and other extractives (% L and O) was calculated using Equation (5):

(5) % Lignin & Other Extractives (% L & O)=100%−(%H+%C)

### 2.4. Strong Acid Hydrolysis of Cellulose

Cellulose from MFS was hydrolyzed using sulfuric acid at varying concentrations (40, 50, and 60 wt. %) and temperatures (30, 40, 50, and 60 °C) for 45 min. The hydrolysis process was carried out using 50 mL glass lab vials with screw caps in a water bath shaker at 250 rpm. The process was carried out explicitly by weighing ~0.5 g of extracted cellulose and then deliberately adding it to the preheated solutions of sulfuric acid (10 mL) under the desired conditions. The mixture was then transferred to a 250 mL Erlenmeyer flask with 80 mL of cold distilled water under a fume hood to quench the solution. Another 20 mL of cold distilled water was used to wash off the remaining residue of the hydrolysis mixture in the glass lab vials. The mixture was then homogenized using a vortex mixer for 10 s and then centrifuged (HSIANGTAI Centrifuge, New Taipei, Taiwan) at 3500 rpm for 30 min. After centrifugation, a layer of supernatant and precipitate was observed in the vials. The supernatants were discarded while the precipitates were then dialyzed using cellulose dialysis tubing (MW14000 Regenerated Cellulose RC Dialysis Bag, Hefei, China) with a 14,000 Da cut-off until a solvent (water) of pH 7 was achieved (7 days). The dialyzed samples were freeze-dried (ilShinbiobase Co., Ltd., Dongducheon, Republic of Korea) at −40 °C for 54 h and weighed [4,7,26].

#### Calculation of Nanocellulose Yield from Maguey Cellulose

The post-hydrolyzed cellulose was designated as the nanocellulose product after the presence of nanoparticles was verified via morphological analysis. The nanocellulose yield (*Y_N_*) was calculated using Equation (6):

(6) $$\%\;Nanocellulose\;yield\;(Y_{N})=\frac{m_{N}}{m_{CCS}}\times 100\;\%$$

where *m_N_* is the mass of nanocellulose (g), and *m_CCS_* is the mass of cellulose sample (g).

Response surface methodology (RSM) through DX-Expert Version 22.0.3 software was administered to analyze the results of the experiment and ensured that the accuracy and sensitivity of the results were carried over. Furthermore, a two-way analysis of variance (ANOVA) without replication was also used to analyze nanocellulose yields and to statistically evaluate the impact of temperature and acid concentration as independent variables on nanocellulose yield. This evaluation assesses the statistical significance of these effects, ruling out chance occurrences.

### 2.5. Characterization

#### 2.5.1. Morphological Analysis via Scanning Electron Microscopy and Transmission Electron Microscopy

Field emission scanning electron microscope (FE-SEM) analyses were conducted on raw maguey fibers, cellulose, and post-hydrolyzed cellulose. FE-SEM was carried out for both maguey raw fibers and post-hydrolyzed cellulose and analyzed under a Zeiss microscope, Jena, Germany at 5.0 kV; the FE-SEM analysis of the extracted cellulose was analyzed uncoated with benchtop SEM model JCM-7000, JEOL Ltd., Tokyo, Japan at 10.0 kV, and another FE-SEM for post-hydrolyzed cellulose was analyzed using JEOL JSM-IT800, JEOL Ltd., Tokyo, Japan at 15.0 kV. For the raw MFS, the samples were coated with gold, with a 3 nm thickness, using a sputtering machine. The dimensions of the nanoparticles were manually measured using AutoCAD Version S.51.0.0 software.

Moreover, transmission electron microscopy (TEM) analysis was carried on the post-hydrolyzed cellulose using JEOL JEM-2100F, JEOL Ltd., Tokyo, Japan with 200 accelerating voltages in a 2 µm and 50 nm micron marker. Information on the inner structure of the post-hydrolyzed cellulose sample was analyzed. The dimensions of the nanoparticles were also manually measured using AutoCAD Version S.51.0.0 software.

#### 2.5.2. Fourier Transform Infrared Spectroscopy (FTIR)

The FTIR analyses for the raw MFS, cellulose, and post-hydrolyzed cellulose were performed using IRAffinnity-1s, SHIMADZU, Kyoto, Japan, through zinc selenide attenuated total reflectance (ZnSe, ATR, SHIMADZU, Kyoto, Japan) in the range of 600 to 4000 cm^−1^. The samples were finely powdered using an electric rotary-type mill before the analysis.

#### 2.5.3. Zeta Potential Analysis

The zeta potential of the nanocellulose was measured using the SZ-100, HORIBA, Kyoto, Japan instrument. The measurement was performed by suspending approximately 0.05 g of the nanocellulose sample in 50 mL of water, followed by sonication for 3 h to ensure uniform dispersion. The instrument was set to an electrode voltage of 3.3 V and a conductivity of 0.285 mS/cm. The measurement was performed at constant temperature of 25 °C and dispersion medium viscosity of 0.895 mPa.s with a range from −200 mV to 200 mV.

#### 2.5.4. X-ray Diffraction (XRD)

The XRD analysis was performed using a LabX-6000, SHIMADZU, Kyoto, Japan X-ray diffractometer with an aluminum plate sample holder (25 mm diameter and 1 mm depth) at 40.0 kV and 30.0 mA with Cu Kα radiation (1.5148 Å). The post-hydrolyzed cellulose samples were finely powdered for 1 min using an 800Y Kankeirr, Guangzhou, China electric rotary-type mill with sharp steel blades and sent for analysis. The post-hydrolyzed cellulose sample was analyzed without any further processing of the raw data. The data were acquired in a 2θ range from 2° to 70°. The detailed description of the XRD analysis profile data can be accessed in Section S.IV.4. in the Supplementary Material. The crystallinity index ($I_{C}$) of the nanocellulose from maguey fibers obtained via a series of chemical treatments was calculated using the XRD peak height method by Segal et al. (1959), as shown in Equation (7) [27]:

(7) $$I_{C}=\frac{I_{200}-I_{am}}{I_{200}}\times 100\;\%$$

where *I*_200_ is the maximum intensity of diffraction of the (200) lattice peak around 22° to 23° and *I_am_* is the minimum intensity between 18° and 19°, representing the amorphous material in this empirical equation.

#### 2.5.5. Thermogravimetric Analysis (TGA)

The TGA of the post-hydrolyzed cellulose was performed using a Simultaneous Thermal Analyzer (STA) 6000, PerkinElmer, Waltham, MA, USA measuring cell to characterize the thermal stability of the nanocellulose obtained from the maguey fibers. Approximately 13.549 mg of sample was held in a ceramic crucible and heated from 30 °C to 600 °C at a heating rate of 10 °C/min. The measurement was performed under a nitrogen atmosphere at 20 mL/min from 30 °C to 600 °C.

## 3. Results and Discussion

### 3.1. Removal of Non-Cellulosic Components of Maguey Fiber by Alkali Treatment and Bleaching

#### 3.1.1. Cellulose Yield from Maguey Fiber

MFS presents a cellulose yield of 53.56% after alkali treatment and bleaching processes. This corresponds well with the cellulose yield of other *Agave* plant species and competes with other natural fibers that underwent similar processes, as presented in Table 1. MFS has a relatively higher cellulose yield than other natural fibers with similar isolation processes. This implies the significance of MFS as a better alternative cellulose source that is locally available.

#### 3.1.2. Holocellulose, α-Cellulose, Hemicellulose, and Lignin and Other Extractive Contents

After removing non-cellulosic components via alkali treatment and bleaching, the bleached MFS was composed of α-cellulose and traces of hemicellulose, lignin, and other extractives. These components were determined via percent composition following various determination procedures. The bleached product comprised holocellulose, which refers to α-cellulose and hemicellulose combined, and lignin and other extractives. The holocellulose content was determined to be 92.41%. The composition of the bleached MFS is presented in Table 1. Detailed calculations of the holocellulose, α-cellulose, hemicellulose, and lignin and other extractive contents can be seen in Section II of the Supplementary Material.

As a result of alkali treatment and bleaching carried out on the MFS, bleached MFS with α-cellulose content of 89.45% is presented in Table 1. This α-cellulose content is competitive with other reported α-cellulose contents of other *Agave* plant species and is relatively higher than that of other natural fibers. The bleached MFS has a hemicellulose content of 2.96%, which is lower than that of other reported hemicellulose and has a lignin content of 7.59%.

The hemicellulose content of the bleached MFS corresponds well with the alkali treatment process. The repetition of the alkali treatment process further removed more hemicellulose in the MFS, leading to lower hemicellulose content compared with those under processes without repeated alkali treatment. Moreover, the lignin content of the bleached MFS was relatively higher, corresponding with the bleaching process carried out on MFS. The bleaching process directly affected the lignin composition, which reacted as lignin chloride [7]. The alkali-treated maguey fiber was bleached from the preheated temperature to room temperature in a shorter period than other reported bleached fibers. The significant amount of α-cellulose produced indicates that maguey fiber is an excellent alternative cellulose source for nanocellulose production.

### 3.2. Strong Acid Hydrolysis of Cellulose

#### 3.2.1. Nanocellulose Yield from Strong Acid Hydrolysis of Cellulose

**Figure 1:**
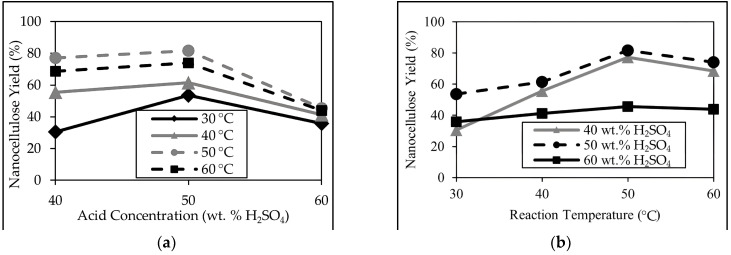
Graph of average nanocellulose yield with respect to (**a**) varying acid concentrations and (**b**) reaction temperatures.

The nanocellulose yield from strong acid hydrolysis of cellulose is analyzed with respect to the varying conditions of two independent variables, namely acid concentrations and reaction temperatures. The relationship between the two independent variables with the nanocellulose yield varies as presented in Figure 1.

The acid hydrolyses at all acid concentrations demonstrate a maximum nanocellulose yield along with a 50 wt. % acid concentration while the acid hydrolyses at all reaction temperatures depict a maximum nanocellulose yield at 50 °C, as shown in Figure 1a and Figure 1b, respectively.

Under ideal acid hydrolysis conditions, the amorphous regions of the cellulose fibrils are hydrolyzed and removed by the acid while maintaining the crystalline areas of the cellulose fibrils. However, at temperatures below 50 °C and at an acid concentration of 50 wt. % H_2_SO_4_, cellulose is under-degraded due to the inability of these conditions to solubilize and degrade the amorphous regions, preventing the formation of nanocrystalline cellulose. On the other hand, under hydrolysis conditions exceeding 50 °C and a 50 wt. % H_2_SO_4_ concentration, cellulose is over-degraded, causing the decomposition of both crystalline and amorphous regions, resulting in a lower yield.

The acid hydrolysis at 50 °C and a 50 wt. % H_2_SO_4_ concentration has the highest observed yield of 81.58 ± 0.36%, while hydrolysis at the temperature of 30 °C and a 40 wt. % H_2_SO_4_ concentration has the lowest observed yield of 30.41 ± 1.71%.

The observed variance in nanocellulose yields is analyzed via a two-way analysis of variance (ANOVA), and simple main effects analysis concludes, at a 95% level of confidence, that both temperature and acid concentration have statistically significant effects on nanocellulose yield.

Moreover, undergoing a multilevel categoric full factorial design type via the response surface methodology of DX-Design Version 22.0.3 software, the analysis obtained a final equation, Equation (8):

(8) $$\begin{array}{c} Nanocellulose\;Yield\\ \;\;\;\;\;\;=57.07-1.56A+12.20A^{2}-9.27B-9.88B^{2}+13.36B^{3}-10.62AB\\ \;\;\;\;\;\;+3.37A^{2}B-3.73AB^{2}-0.9168A^{2}B^{2}+7.27AB^{3}-0.7924A^{2}B^{3} \end{array}$$

where A refers to the acid concentration and B is the reaction temperature. The equation can be used to make predictions about the response for given levels of each factor. By default, the high levels of the factors are coded as +1 and the low levels are coded as −1. The coded equation is useful for identifying the relative impact of the factors by comparing the factor coefficients. A very detailed discussion on ANOVA and RSM results can be found in Section III of the Supplementary Material.

The nanocellulose produced from maguey fibers was compared to other nanocellulose obtained from other *Agave* species and other non-wood nanocellulose sources with similar process parameters together with the nanocellulose characteristics including morphological dimensions, crystallinity, and thermal stability, as shown in Table 2. The comparative analysis of nanocellulose yield and characteristics against other *Agave* and non-wood nanocellulose sources exhibits competitive results. Notably, the yield from the produced nanocellulose is within the range of that of *Agave tequilana* Weber var. Azul based on the cellulose sample demonstrating levels similar to the reported nanocellulose yield. Furthermore, the yield of the nanocellulose produced in this study appears relatively higher when compared with that of other reported nanocellulose based on the fiber sample shown in Table 2. This observation implies that maguey fiber is a significant alternative source for nanocellulose.

Moreover, the morphological dimensions of the produced nanocellulose coincide with those of other reported nanocellulose. This morphological similarity displays the consistency of maguey nanocellulose with the broader variety of non-wood cellulose sources. In addition, the crystallinity index and thermal stability of the produced nanocellulose demonstrate competitive qualities that further enhance its significance and potential applications. 

#### 3.2.2. Morphological Analysis of Maguey Fiber, Cellulose, and Post-Hydrolyzed Cellulose

**Figure 2:**
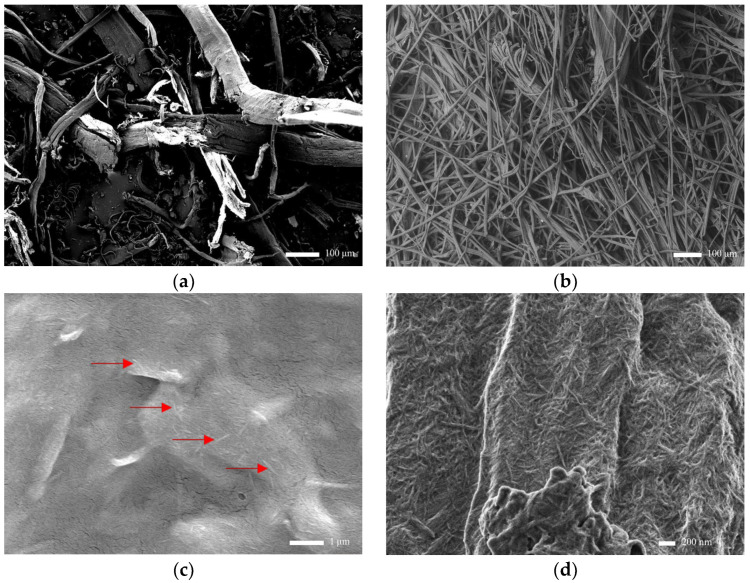
Field emission scanning electron microscopy (FE-SEM) micrographs of (**a**) raw maguey fiber sample (MFS), (**b**) extracted cellulose, (**c**) sonicated post-hydrolyzed cellulose (dispersed nanocellulose indicated with red arrows), and (**d**) non-sonicated post-hydrolyzed cellulose.

The FE-SEM micrographs of the maguey fiber, cellulose, and post-hydrolyzed cellulose are presented in Figure 2. The following figures show a change in the maguey fiber’s morphology after going through various chemical processes: alkali treatment, bleaching, and acid hydrolysis.

The FE-SEM micrograph of the raw maguey fiber shown in Figure 2 has a length range from 400 μm to 700 μm and a diameter range from 90 μm to 110 μm. The fiber surface is uneven and rough, and has an irregular shape, implying the presence of impurities that ought to be hemicelluloses, lignin, and other extractives.

Meanwhile, the FE-SEM micrograph of the extracted cellulose depicts an apparent change in the morphology of the maguey fibers. A smoother surface and narrower microscale cellulose strands can be observed as it underwent alkali treatment and bleaching processes. However, there are inconsistent structures aside from the fibrous structures along the cellulose strands, implying that there are still impurities, such as hemicellulose and lignin. The length and diameter of the cellulose strands range from 23 μm to 256 μm and 5 μm to 13 μm, respectively.

Moreover, the micrograph of the post-hydrolyzed cellulose produced under the best hydrolysis conditions based on the highest yield (at 50 °C using 50 wt. % H_2_SO_4_) shows an apparent change in morphology as the extracted cellulose underwent the acid hydrolysis process. Figure 2c presents the sonicated post-hydrolyzed cellulose sample prior to FE-SEM analysis, displaying a dispersed nanocellulose in the micrograph, as indicated with the red arrows. Small rod-like structures at the nanoscale are scattered throughout the image, observed at an approximate magnification of 10,000 times. On the other hand, in Figure 2d, the FE-SEM image without the sonication of the post-hydrolyzed cellulose sample displays agglomerated nanocellulose in the micrograph with a magnification of exactly 30,000 times. The length and diameter of the nanoparticles range from 72 nm to 866 nm and 8 nm to 75 nm, respectively. With the length of the nanoparticles exceeding 100 nm and differing significantly from their diameter, the nanoparticles produced can also be considered nanofibrillar. However, nanofiber is mostly associated with a length of several micrometers; thus, the produced nanoparticles will be acknowledged as nanocellulose. A very detailed characterization of nanocellulose can be seen in Section IV of the Supplementary Material.

**Figure 3:**
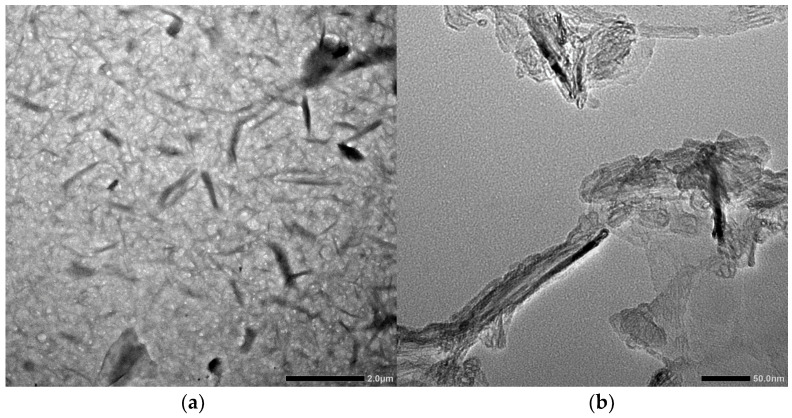
Transmission electron microscopy (TEM) micrograph of post-hydrolyzed cellulose at (**a**) 2.0 µm scale and (**b**) 50.0 nm scale.

TEM analysis was also carried out to examine the morphology of the post-hydrolyzed cellulose, which provides an excellent resolution, enabling a closer look at the structure and size of the sonicated post-hydrolyzed cellulose sample, as shown in Figure 3. Figure 3a clearly shows rod-like structures similar to what is observed in Figure 2c. The length and diameter coincide with the manually measured structures in the SEM micrographs. The measured dimensions present an average length of 491.57 nm and an average diameter of 69.99 nm. In Figure 3b, the inner structure of the nanoparticles is solid, which corresponds to the formation of a crystalline rod-like structure, attributed to the cleavage of the amorphous region of the cellulose during acid hydrolysis [39].

#### 3.2.3. Fourier Transform Infrared (FTIR) Spectroscopy Analysis of Maguey Fiber, Cellulose, Nanocellulose

**Figure 4:**
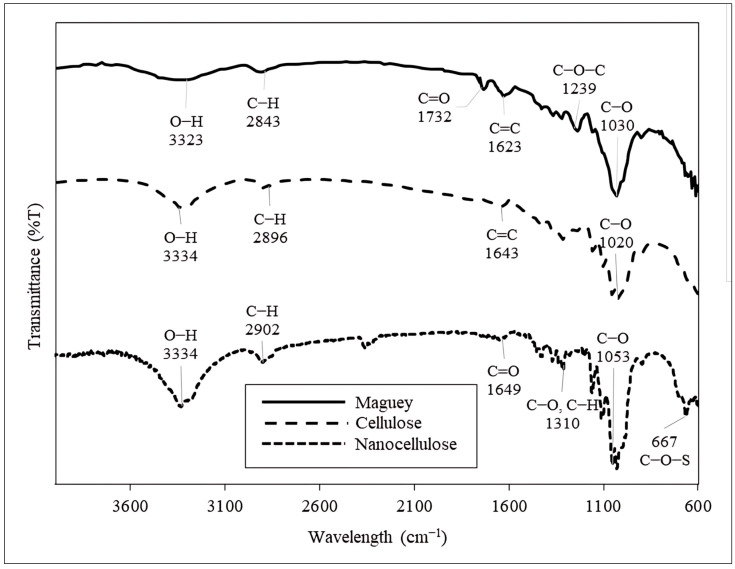
Fourier transform infrared spectroscopy (FTIR) spectrum of raw maguey fiber (data were generated using OriginPro).

The results of the FTIR analysis for the raw maguey fiber, cellulose, and nanocellulose are shown in Figure 4. The figure presents infrared transmittance, shown in peaks, for the raw maguey fiber, cellulose, and nanocellulose spectrum. The appearance and absence of certain peaks indicate changes in the chemical composition of the fibers as they underwent alkali treatment, bleaching, and acid hydrolysis processes. The actual spectrum of the samples can be seen in Section S.IV.3. in the Supplementary Material.

The raw maguey fiber spectrum shows peaks at 1732.94 cm^−1^ and 1623.69 cm^−1^, indicating the presence of C=O and C=C functional groups, respectively [40]. The C=O stretching likely refers to the carbonyl, carboxyl, and acetyl groups corresponding to the presence of cellulose and hemicellulose. Meanwhile, the C=C stretching corresponds to the presence of lignin. Other peaks occur at 1239.30 cm^−1^, 1030.79 cm^−1^, and 2843.29 cm^−1^, and a broad-shallow peak occurs in the 3100–3600 cm^−1^ range, associated with the presence of cellulose and represent aryl-alkyl ether in lignin, primary alcohol in hemicellulose, hydrocarbons, and the hydroxyl stretching vibrations, respectively [41].

The peaks of the raw maguey fiber spectrum appear to be of a lower intensity than the peaks of the cellulose and nanocellulose spectra. The resulting peak of an untreated fiber is influenced by several factors, including different surface conditions due to the presence of impurities. The chemical bond under different surface conditions will absorb varying intensities and frequencies, which likely causes lower absorption of infrared radiation, resulting in a lower intensity of the spectrum peaks [42].

On the other hand, the cellulose spectrum shows the result of the alkali-treated and bleached maguey fibers. The broad peak at 3334.88 cm^−1^ and small peak at 2896.96 cm^−1^ are attributed to the O−H stretching vibration group of cellulose and C−H stretching, respectively. Moreover, the peak at 1643.80 cm^−1^ in this spectrum indicates the presence of a C=C functional group attributed to lignin [43]. The lack of C=O vibrations indicates the effectiveness of the alkali treatment and bleaching processes, as it implies the removal of most hemicellulose.

The spectrum of the nanocellulose produced from maguey fiber exhibits a broad peak at 3334.92 cm^−1^, which is attributed to the O−H stretching vibration and intermolecular hydrogen bonding. The O−H vibration peaks exhibit a correlation with hydrolysis, as prolonged hydrolysis results in a deeper peak within this range [44]. Thus, the change in peak depth is observable from the raw maguey fiber to the nanocellulose sample. Additionally, a peak at 2902.87 cm^−1^ is associated with C−H bending, indicating the enhanced exposure and sharpening of the cellulose components [44,45,46].

Furthermore, an observable peak at 1649.14 cm^−1^ is attributed to the carboxyl groups corresponding to the presence of cellulose (higher intensity peaks), hemicellulose, and lignin (lower intensity peaks), while smaller vibrations (<1315.45 cm^−1^) is attributed to the bending vibration of C−O of secondary alcohols and aromatic C−H bonds in a polysaccharide structure [47]. However, the absence of the C=O attributed to hemicellulose and C=C attributed to lignin, which are commonly found in most untreated fibers, implies the removal of most hemicellulose and lignin during the alkali treatment, bleaching, and the acid hydrolysis process. In the fingerprint region, the peak at 667.37 cm^−1^ is attributed to C−O−S bending, indicating a sulfation peak attributed to the hydrolysis process or the reaction of sulfuric acid [48].

#### 3.2.4. Zeta Potential Analysis of Nanocellulose

The zeta potential of the nanocellulose was measured to evaluate its surface charge and the stability of the nanocellulose suspension. The nanocellulose displays a highly negative surface charge with a mean zeta potential value of −37.3 mV. This negative zeta potential was aided by the negative electrostatic layers formed on the nanocellulose due to the grafting of sulfate groups during sulfuric acid hydrolysis that link to the nanocellulose chains [49]. Nanocellulose with a negative zeta potential is highly preferred as it can resist aggregation in a colloidal environment, leading to a wider distribution [49]. The reported zeta potential value presents a more stable dispersed state compared with values between −15 and 15 mV where agglomeration forms due to the lack of necessary charges for a repelling effect [49]. This result implies that the produced nanocellulose would lead to a uniform nanocellulose suspension due to the strong electrostatic repulsive forces [49,50]. The measurement results can be obtained from Section S.IV.4 in the Supplementary Material.

#### 3.2.5. X-ray Diffraction Analysis of Nanocellulose

**Figure 5:**
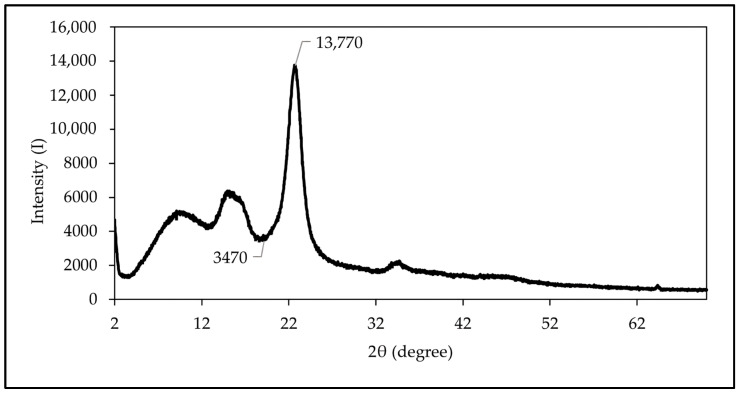
X-ray diffraction (XRD) patterns of nanocellulose from maguey fibers.

Figure 5 shows the X-ray diffraction pattern of the nanocellulose from maguey fiber. The diffraction pattern showed peaks around 2θ = 15.52° and 22.64°, which indicate a typical cellulose I structure [7,15]. The high peak exhibited a higher crystallinity index, indicating the effective removal of amorphous regions during the chemical treatments. The crystallinity index refers to the order of crystallinity of the material rather than the crystallinity of crystalline regions. A higher crystallinity value is mainly attributed to removal of the amorphous constituents during acid hydrolysis.

In Figure 5, the crystallinity of the sample implies the presence of a sharp peak. The height of the highest diffraction peak represents the amount of crystalline material, while the amorphous material is represented by the height of the minimum intensity between the major peaks. The crystallinity of the nanocellulose increases when the lignin, hemicellulose, and other amorphous components are almost completely removed during the series of chemical treatments [48].

The method of Segal et al. (1959) is used to determine the crystallinity index of maguey fibers, known as the XRD peak height method [27]. This method examined the changes in the XRD spectra during the decrystallization of nanocellulose from maguey fiber using a series of chemical treatments. The crystallinity index (I_C_) was determined from the maximum (I_200_) and minimum (I_am_) intensity of the diffraction, which signify the crystallinity and the amorphous material peaks at 22° to 23° and 18° to 19° for a 2θ diffraction angle, respectively. The crystallinity index of the nanocellulose from maguey fiber was found to be 74.80%, while the crystallinity index of some of the nanocellulose from various natural fibers was ~64–82% [7,15,28,38]. In that case, the calculated crystallinity index was within the range of the crystallinity index of nanocellulose among the natural fibers. This high value of the crystallinity index is attributed to the elimination of amorphous constituents during the chemical treatments.

#### 3.2.6. Thermogravimetric Analysis of Nanocellulose

The thermal decomposition parameters of the obtained nanocellulose sample from the maguey fibers were determined from the thermogravimetric–differential thermogravimetric (TG–DTG) of the nanocellulose sample in solid white powder form.

**Figure 6:**
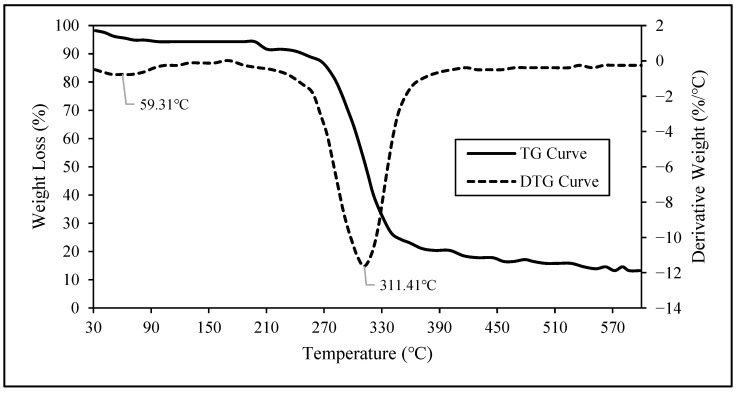
Thermogravimetric (TG) and differential thermogravimetric (DTG) curves of nanocellulose from maguey fibers.

Evident weight loss and corresponding peak temperatures are observed in the resulting TG and DTG curves of the nanocellulose sample, presented in Figure 6. The TG curve illustrates how the weight of the post-hydrolyzed cellulose changes with temperature while the DTG curve indicates the point at which weight loss is most apparent.

Based on the DTG curve presented in Figure 6, it is evident that within the temperature range of 30.00 °C to 129.13 °C, the peak temperature of 59.31 °C corresponds to 4.72% weight loss, primarily attributed to the evaporation of moisture during the heating process. During the heating in the temperature range of 129.13 °C to 427.81 °C, the inflection point or the first derivative peak temperature, indicating that the point of the greatest rate of change on the weight loss curve was at 311.41 °C, where 81.22% weight loss occurred. This weight loss is attributed to the alpha-cellulose degradation at this heating temperature [7]. The residual weight at the final temperature of 600 °C is found to be 14.06% of the initial weight of the sample. A fraction of this residual weight of post-hydrolyzed cellulose is attributed to the remaining lignin. Lignin has a slow degradation rate, and, when it is present in the sample, a higher residual weight is expected [7]. The remaining fraction of the residual weight can be attributed to the remaining ash present, the thermal decomposition of which occurs above the final temperature. A detailed report of this analysis can be found in Section S.IV.6 in the Supplementary Material.

## 4. Conclusions

Cellulose was successfully obtained from maguey (*Agave cantala*) fibers via alkali treatment and bleaching processes with a yield of 53.56%. The high cellulose yield of maguey fiber demonstrates an efficient process and embodies promising qualities in terms of sustainability and cost-effectiveness. This high yield not only enhances productivity and resource efficiency but also contributes significantly to sustainable nanocellulose production. Maximizing output through an efficient process leads to reductions in cost and resource conservation. The availability of maguey fiber as a local source of cellulose also has economic advantages in terms of transportation, resource acquisition, and flexible production. Additionally, local availability can also contribute to lower carbon emissions and other environmental impacts, leading to more sustainable production.

Cellulose nanocrystals were also successfully produced from maguey cellulose via strong acid hydrolysis, as proven by the nanoscale dimensions measured in the morphological analysis and the crystallinity index measured via XRD analysis. Variations in the acid concentration and reaction temperatures of the acid hydrolysis of cellulose from maguey fibers show significant effects on the yield of nanocellulose produced, as supported by the two-way ANOVA.

This study found that the acid hydrolysis conditions that provided the highest nanocellulose yield are 50 °C and a 50 wt. % H_2_SO_4_ concentration. FE-SEM and TEM analyses showed that the maguey fibers were successfully disintegrated from 90–110 µm to 8–75 nm in diameter and from 400–700 µm to 72–866 nm in length. FTIR analysis showed that the chemical composition of the maguey fibers changed from that of raw fibers to that of nanocellulose, indicating the removal of most impurities. The highly negative surface charge of the nanocellulose with a mean zeta potential value of −37.3 mV indicates a more stable dispersed state of the nanocellulose suspension due to the strong electrostatic repulsion. Through XRD analysis, the crystallinity index of the nanocellulose was found to be 74.80%. On the other hand, thermogravimetric analysis of the nanocellulose indicated that the point of the greatest rate of thermal degradation occurred at 311.41 °C, which is attributed to cellulose degradation.

The nanocellulose produced from maguey fiber embodies desirable properties, thereby revealing the potential of maguey fiber as an alternative source of nanocellulose. This offers an efficient solution for diverse practical applications such as in polymers due to its competitive crystallinity index and stable dispersed state. Other essential qualities of nanocellulose include biocompatibility, sustainability, and cost-effectiveness, making it an ideal candidate for advanced material applications.

## Figures and Tables

**Table 1:** Cellulose yield and composition of various *Agave* plant species and other natural fibers.

Fiber Sources	Process Parameters		Lignocellulose Yield, %	Components, %			Reference
	Alkali Treatment	Bleaching		α-Cellulose	Hemicellulose	Lignin and Other Extractives	
*Agave* *cantala*	4 wt. % NaOH, 75 °C, 2 h *	5 *v*/*v* % NaClO, 70 °C to room temp., 2 h	53.56	89.45 ± 2.64	2.96 ± 0.13	7.59 ± 2.77	Present study
*Agave americana*	2–4 *w*/*v* % NaOH, 80 °C, 2 h	2 *w*/*v* % NaClO_2_, 80 °C, 4 h	65.00 ± 2.00	90.00 ± 5.00	9.00 ± 5.00	0.04 ± 0.10	[28]
*Agave* *angustifolia*	4% NaOH, 70–80 °C, 2 h *	1.7 *w*/*v* % NaClO_2_, 70–80 °C, 4 h	67.01 ± 0.15	97.31 ± 0.02	3.14 ± 0.35	0.23 ± 0.04	[7]
*Agave * *gigantea*	5 *w*/*v* % NaOH, 80 °C, 2 h	1.7 wt. % NaClO_2_, 80 °C, 1 h *	n.a.	89.39	3.73	0.53	[29]
Apple pomace	6% NaOH, 60 °C, 30 min	1% NaClO, 95 °C, 1 h	33.17	84.72	n.a.	n.a.	[26]
Banana sheath	2% NaOH, room temp., 6 h	5% NaClO_2_, 50 °C, 1 h	28.40	46.50	9.34	11.62	[30]
Pineapple leaf	2 *w*/*v* % NaOH	4 *w*/*v* % NaClO, 85 ± 5 °C, 4 h	70.90 ± 1.10	85.53 ± 2.30	0.30 ± 0.90	0.40 ± 0.30	[31]
Sugar palm	10 *w*/*w* % NaOH, 150 °C, 2 h	15% H_2_O_2_, 60 °C, 1.5 h	n.a.	86.99 ± 1.98	9.95 ± 0.87	2.90 ± 1.38	[32]

n.a.: not available; * repeated twice.

**Table 2:** Nanocellulose yield and characteristics from various *Agave* species and other natural fibers.

Fiber Sources	Process Parameters	Nanocellulose Yield, %	Characteristics				Reference
	Acid Hydrolysis		Diameter (nm)	Length (nm)	Crystallinity Index (%)	Thermal Stability (°C)	
*Agave cantala*	50 wt. % H_2_SO_4_, 50 °C, 45 min	81.58 ± 0.36 (43.50–43.89 *)	8–75	72–866	74.80	311.41	Present study
*Agave americana*	70% HNO_3_ and 80% CH_3_COOH, 100 °C, 30 min	27.0 *	n.a	18.2 ± 10.14	64.11	374.70	[28]
*Agave* *angustifolia*	60 wt. % H_2_SO_4_, 45 °C, 45 min	n.a	8–15	170–500	82.40	361	[7]
*Agave sisalana*	55 wt. % H_2_SO_4_, 45–60 °C, 20–30 min	n.a	5.9 ± 1.0–10.5 ± 2.9	177 ± 56–433 ± 132	n.a	n.a	[33]
*Agave tequilana*	65 wt. % H_2_SO_4_, 50 °C, 60 min	n.a	11 ± 4	323 ± 113	71	324	[34]
*Agave tequilana* Weber var. Azul	60–65 wt. % H_2_SO_4_, 40–60 °C, 40–70 min	4.2–96	8.6–9.1	216–829	88.4–90.1	n.a	[35]
Apple pomace	45% H_2_SO_4_, 50 °C, 45 min	n.a	7.9 ± 1.25	28 ± 2.03	78	187	[26]
*Acacia fornesiana* L. Willd	60–65 wt. % H_2_SO_4_, 45–55 °C, 45–65 min	n.a	n.a	100–260	n.a	n.a	[36]
Banana pseudostem	11 M H_2_SO_4_, 50 °C, 30–240 min	10 *	13 ± 4– 19 ± 6	319 ± 68–466 ± 159	69–74	n.a	[37]
Barley	65 wt. % H_2_SO_4_, 50 °C, 60 min	n.a	10 ± 4	329 ± 123	66	357	[34]
Sugarcane bagasse	60 *w*/*v* % H_2_SO_4_, 50 °C, 5 h	n.a	35	170	n.a	345	[38]

n.a: not available; * based on fiber.

## Data Availability

The data presented in this study are available in the Supplementary Material.

## Supplementary Materials

The following supporting information can be downloaded at: https://www.mdpi.com/article/10.3390/polym16101312/s1, contains data gathered from SEM micrographs analysis, raw data from FTIR analysis, Zeta Potential analysis, XRD analysis profile data, and TGA data. Supplementary I: Characterization of the maguey fiber sample; Supplementary II: Determination of cellulose, hemicellulose, and lignin and other extractive contents; Supplementary III: Raw data and analysis via Response Surface Methodology and two-factor ANOVA of nanocellulose yield from maguey fiber; Supplementary IV: Characterization of nanocellulose.

## References

[1] Norizan M.N., Shazleen S.S., Alias A.H., Sabaruddin F.A., Asyraf M.R.M., Zainudin E.S., Abdullah N., Samsudin M.S., Kamarudin S.H., Norrrahim M.N.F. (2022). Nanocellulose-Based Nanocomposites for Sustainable Applications: A Review. Nanomaterials.

[2] Chen P., Re G., Berglund L., Wohlert J. (2020). Surface Modification Effects on Nanocellulose—Molecular Dynamics Simulations Using Umbrella Sampling and Computational Alchemy. J. Mater. Chem. A.

[3] Phanthong P., Reubroycharoen P., Hao X., Xu G., Abudula A., Guan G. (2018). Nanocellulose: Extraction and Application. Carbon Resour. Convers..

[4] Trache D., Tarchoun A.F., Derradji M., Hamidon T.S., Masruchin N., Brosse N., Hussin M.H. (2020). Nanocellulose: From Fundamentals to Advanced Applications. Front. Chem..

[5] Abitbol T., Rivkin A., Cao Y., Nevo Y., Abraham E., Ben-Shalom T., Lapidot S., Shoseyov O. (2016). Nanocellulose, a Tiny Fiber with Huge Applications. Curr. Opin. Biotechnol..

[6] Balea A., Fuente E., Concepcion Monte M., Merayo N., Campano C., Negro C., Blanco A. (2020). Industrial Application of Nanocelluloses in Papermaking: A Review of Challenges, Technical Solutions, and Market Perspectives. Molecules.

[7] Rosli N.A., Ahmad I., Abdullah I. (2013). Isolation and Characterization of Cellulose Nanocrystals from *Agave angustifolia* Fibre. BioRes.

[8] George J., Sabapathi S.N. (2015). Cellulose Nanocrystals: Synthesis, Functional Properties, and Applications. Nanotechnol. Sci. Appl..

[9] Baskakov S.A., Baskakova Y.V., Kabachkov E.N., Kichigina G.A., Kushch P.P., Kiryukhin D.P., Krasnikova S.S., Badamshina E.R., Vasil’ev S.G., Soldatenkov T.A. (2022). Cellulose from Annual Plants and Its Use for the Production of the Films Hydrophobized with Tetrafluoroethylene Telomers. Molecules.

[10] Fauzee S.N., Othaman R. (2013). Extraction and Dissolution of Cellulose from Nypa Fruit Husk for Nanofibers Fabrication. AIP Conf. Proc..

[11] Mustikaningrum M., Cahyono R.B., Yuliansyah A.T. (2021). Effect of NaOH Concentration in Alkaline Treatment Process for Producing Nano Crystal Cellulose-Based Biosorbent for Methylene Blue. IOP Conf. Ser. Mater. Sci. Eng..

[12] Wang T., Zhao Y. (2021). Optimization of Bleaching Process for Cellulose Extraction from Apple and Kale Pomace and Evaluation of Their Potentials as Film Forming Materials. Carbohydr. Polym..

[13] Li M., Liu X., Lv K., Sun J., Dai C., Liao B., Liu C., Mei C., Wu Q., Hubbe M. (2023). Cellulose nanomaterials in oil and gas industry: Current status and future perspectives. Prog. Mater. Sci..

[14] Kandhola G., Djioleu A., Rajan K., Labbé N., Sakon J., Carrier D.J., Kim J.W. (2020). Maximizing Production of Cellulose Nanocrystals and Nanofibers from Pre-Extracted Loblolly Pine Kraft Pulp: A Response Surface Approach. Bioresour. Bioprocess..

[15] Huntley C.J., Crews K.D., Abdalla M.A., Russell A.E., Curry M.L. (2015). Influence of Strong Acid Hydrolysis Processing on the Thermal Stability and Crystallinity of Cellulose Isolated from Wheat Straw. Int. J. Chem. Eng..

[16] Wulandari W.T., Rochliadi A., Arcana I.M. (2016). Nanocellulose Prepared by Acid Hydrolysis of Isolated Cellulose from Sugarcane Bagasse. IOP Conf. Ser. Mater. Sci. Eng..

[17] Oriez V., Peydecastaing J., Pontalier P.Y. (2019). Lignocellulosic Biomass Fractionation by Mineral Acids and Resulting Extract Purification Processes: Conditions, Yields, and Purities. Molecules.

[18] Bacha E.G. (2022). Response Surface Methodology Modeling, Experimental Validation, and Optimization of Acid Hydrolysis Process Parameters for Nanocellulose Extraction. S. Afr. J. Chem. Eng..

[19] Hulle A., Kadole P., Katkar P. (2015). *Agave americana* Leaf Fibers. Fibers.

[20] Kolte P.P., Kolte M.P.P., Daberao M.A.M. (2012). *Agave americana*: The Natural Leaf Fiber. Text. Rev..

[21] Anwar Hossain M., Abul Hossain Head M., Abdullah AI-Mamun Head S.M., Rahman M., Shahabuddin M., Shariful Alam Treasurer K. (2009). Isolation and Characterisation of *Agave Cantala* (Dakatia) Fibre. J. Sci. Technol..

[22] Palacios Hinestroza H., Hernández Diaz J.A., Esquivel Alfaro M., Toriz G., Rojas O.J., Sulbarán-Rangel B.C. (2019). Isolation and Characterization of Nanofibrillar Cellulose from *Agave tequilana* Weber Bagasse. Adv. Mater. Sci. Eng..

[23] Ortega Z., Castellano J., Suárez L., Paz R., Díaz N., Benítez A.N., Marrero M.D. (2019). Characterization of *Agave americana* L. Plant as Potential Source of Fibres for Composites Obtaining. SN Appl. Sci..

[24] Yang X., Berglund L.A. (2021). Structural and Ecofriendly Holocellulose Materials from Wood: Microscale Fibers and Nanoscale Fibrils. Adv. Mater..

[25] Trindade W.G., Hoareau W., Megiatto J.D., Razera I.A.T., Castellan A., Frollini E. (2005). Thermoset Phenolic Matrices Reinforced with Unmodified and Surface-Grafted Furfuryl Alcohol Sugar Cane Bagasse and Curaua Fibers: Properties of Fibers and Composites. Biomacromolecules.

[26] Melikoğlu A.Y., Bilek S.E., Cesur S. (2019). Optimum Alkaline Treatment Parameters for the Extraction of Cellulose and Production of Cellulose Nanocrystals from Apple Pomace. Carbohydr. Polym..

[27] Segal L., Creely J.J., Martin A.E., Conrad C.M. (1959). An Empirical Method for Estimating the Degree of Crystallinity of Native Cellulose Using the X-Ray Diffractometer. Text. Res. J..

[28] Krishnadev P., Subramanian K.S., Janavi G.J., Ganapathy S., Lakshmanan A. (2020). Synthesis and Characterization of Nano-Fibrillated Cellulose Derived from Green *Agave americana* L. Fiber. BioRes.

[29] Syafri E., Jamaluddin S.N., Mahardika M., Amanda P., Ilyas R. (2022). Isolation and Characterization of Cellulose Nanofibers from *Agave gigantea* by Chemical-Mechanical Treatment. Int. J. Biol. Macromol..

[30] Subramanian P., Sriramajayam S., Vijayakumary P., Raja K., Reddy M., Research P.G. (2022). Extraction of Cellulose from Banana Sheath and Its Characterization. Pharma Innov. J..

[31] Fareez I.M., Ibrahim N.A., Wan Yaacob W.M.H., Mamat Razali N.A., Jasni A.H., Abdul Aziz F. (2018). Characteristics of Cellulose Extracted from Josapine Pineapple Leaf Fibre after Alkali Treatment Followed by Extensive Bleaching. Cellulose.

[32] Fitriana N.E., Suwanto A., Jatmiko T.H., Mursiti S., Prasetyo D.J. (2020). Cellulose Extraction from Sugar Palm (*Arenga Pinnata*) Fibre by Alkaline and Peroxide Treatments. IOP Conf. Ser. Earth Environ. Sci..

[33] Siqueira G., Tapin-Lingua S., Bras J., Peres D., Dufresne A. (2010). Morphological investigation of nanoparticles obtained from combined mechanical shearing, and enzymatic and acid hydrolysis of sisal fibers. Cellulose.

[34] Espino E., Cakir M., Domenek S., Román-Gutiérrez A.D., Belgacem N., Bras J. (2014). Isolation and characterization of cellulose nanocrystals from industrial by-products of *Agave tequilana* and barley. Ind. Crops Prod..

[35] Gallardo-Sánchez M.A., Diaz-Vidal T., Navarro-Hermosillo A.B., Figueroa-Ochoa E.B., Ramirez Casillas R., Anzaldo Hernández J., Rosales-Rivera L.C., Martinez A., Enríques S., Macías-Balleza E.R. (2021). Optimization of the Obtaining of Cellulose Nanocrystals from *Agave tequilana* Weber Var. Azul Bagasse by Acid Hydrolysis. Nanomaterials.

[36] Ramírez Casillas R., del Carmen López López M., Becerra Aguilar B., Dávalos Olivares F., Satyanarayana K.G. (2019). Preparation and Characterization of Cellulose Nanocrystals using Soluble Grade Cellulose from Acid Hydrolysis of Huizache (*Acacia farnesiana* L. Willd.). BioResources.

[37] Mueller S., Weder C., Foster E.J. (2014). Isolation of cellulose nanocrystals from pseudostems of banana plants. RSC Adv..

[38] Mandal A., Chakrabarty D. (2011). Isolation of nanocellulose from waste sugarcane bagasse (SCB) and its characterization. Carbohydr. Polym..

[39] Ishak W.H.W., Ahmad I., Ramli S., Amin M.C.I.M. (2018). Gamma Irradiation-Assisted Synthesis of Cellulose Nanocrystal-Reinforced Gelatin Hydrogels. Nanomaterials.

[40] IR Spectrum Table & Charts. https://www.sigmaaldrich.com/PH/en/technical-documents/technical-article/analytical-chemistry/photometry-and-reflectometry/ir-spectrum-table.

[41] Lopes J.d.O., Garcia R.A., de Souza N.D. (2018). Infrared Spectroscopy of the Surface of Thermally-Modified Teak Juvenile Wood. Maderas Cienc. Tecnol..

[42] Merlic C., Fam B., The Regents of University of California Problems in NMR and IR Spectroscopy. https://webspectra.chem.ucla.edu/index.html.

[43] Nacos M.K., Katapodis P., Pappas C., Daferera D., Tarantilis P.A., Christakopoulos P., Polissiou M. (2006). Kenaf Xylan—A Source of Biologically Active Acidic Oligosaccharides. Carbohydr. Polym..

[44] Fitriani F., Aprilia S., Arahman N., Bilad M.R., Amin A., Huda N., Roslan J. (2021). Isolation and Characterization of Nanocrystalline Cellulose Isolated from Pineapple Crown Leaf Fiber Agricultural Wastes Using Acid Hydrolysis. Polymers.

[45] Tan X.Y., Lai C.W., Hamid S.B.A. (2015). Facile Preparation of Highly Crystalline Nanocellulose by Using Ionic Liquid. Adv. Mater. Res..

[46] Dos Reis R.R., Effting C., Schackow A. (2023). Cellulose Nanofibrils on Lightweight Mortars for Improvement of the Performance of Cement Systems. Carbohydr. Polym. Technol. Appl..

[47] Orrabalis C., Rodríguez D., Pampillo L.G., Londoño-Calderón C., Trinidad M., Martínez-García R. (2019). Characterization of Nanocellulose Obtained from Cereus Forbesii (a South American Cactus). Mater. Res..

[48] Robles-García M.Á., Del-Toro-Sánchez C.L., Márquez-Ríos E., Barrera-Rodríguez A., Aguilar J., Aguilar J.A., Reynoso-Marín F.J., Ceja I., Dórame-Miranda R., Rodríguez-Félix F. (2018). Nanofibers of Cellulose Bagasse from *Agave tequilana* Weber Var. Azul by Electrospinning: Preparation and Characterization. Carbohydr. Polym..

[49] Whba F., Mohamed F., Idris M., Yahya M. (2023). Surface Modification of Cellulose Nanocrystals (CNCs) to Form a Biocompatible, Stable, and Hydrophilic Substrate for MRI. Appl. Sci..

[50] Jirathampinyo S., Chumchoochart W., Tinoi J. (2023). Integrated Biobased Processes for Nanocellulose Preparation from Rice Straw Cellulose. Processes.

