# Supplementary material for: Production and Characterization of Nanocellulose from Maguey (Agave cantala) Fiber
Source: Polymers (Basel). 2024 May 7;16(10):1312. doi: 10.3390/polym16101312 (PMC11125682; doi:10.3390/polym16101312)
Supplement: Supplementary file 1 [file polymers-16-01312-s001.zip › polymers-2907473-supplementary.pdf]

Supplementary Material

Supplementary I: Characterization of the maguey fiber sample

S.I.1. *Moisture content determination*

The moisture content of the ground MFS was measured by gravimetric analysis, with moisture analysis performed by drying the samples at 105 ± 5 °C. The percent moisture content of the ground MFS was calculated using Equation 2

$$\% \, MC = \frac{m_{fiber} - (m_{dfc} - m_{dc})}{m_{fiber}} \times 100 \, \%$$

where $\% \, MC$ is the percent moisture content of MFS, m_{fiber} is the mass of fiber added to the crucible (g), m_{dfc} is the total mass of fiber and crucible after drying (g), and m_{dc} is the mass of the dried crucible (g). Through the above equation, the moisture content of the ground MFS was calculated to be 5.34 %.

S.I.2. *Particle size determination*

The DMF was cut into 2-2.5 mm length pieces as preparation before being pulverized in a Thomas Model 4 Wiley® Mill with a 1-mm screen plate. Maguey fibers were then sieved for 5 minutes using an ISO 3310-1 Laboratory Test Sieve, Endecotts Ltd., London, England sieve shaker with sieve sizes of 850, 450, 250, and 180 μm.

Table S.I.2. Sieve analysis.

Sieve Size (μm)	Upper & Lower Sieve Size Average, d_i (μm)	Mass of DMF Retained (g)	Mass Fraction of DMF Retained, x_i	Particle Size Diameter (μm)
1000* - 850	925	71.9090	0.3174	293.5950
850 - 450	650	76.1262	0.3360	218.4000
450 - 250	250	26.7962	0.1183	29.5750
250 - 180	180	51.7483	0.2284	41.1120
**Total Mass of MFS**		**226.5797**		
**Mean Particle Size Diameter, d_m**				**582.6820**

*1-mm screen plate of Thomas Model 4 Wiley® Mill

Calculation for Mean Particle Size Diameter

$$d_m = \sum x_i \, d_i$$

Where: x_i is mass fraction of ground DMF retained on a particular sieve size

d_i is the average of the size of top sieve through which the fraction passed and the size of the bottom sieve on which the fraction was retained.

Supplementary II: Determination of cellulose, hemicellulose, and lignin & other extractive content

Table S.II.1. Determination of holocellulose content.

Trials	Mass of sample cellulose (g)	Mass of filter paper (g)	1st Weighing (g)*	2nd Weighing (g)*	% Change in Mass
**1**	1.0034	1.2568	20.5555	20.5167	0.1887
**2**	1.0025	1.2556	29.5003	28.6736	2.8023
**3**	1.0084	1.2523	29.0065	28.4665	1.8617

*Mass (g) = Mass of crucible + Mass of filter paper + Mass of sample

Table S.II.2. Mass of holocellulose content.

Trials	Mass of holocellulose & filter paper (g)	Mass of filter paper (g)	Mass of holocellulose (g)	% Holocellulose
**1**	2.1911	1.2568	0.9343	93.43
**2**	2.2056	1.2556	0.95	95
**3**	2.1534	1.2523	0.9011	90.11

Table S.II.3. Determination of cellulose content.

Trials	Mass of sample holocellulose (g)	Mass of filter paper (g)	1st Weighing (g)*	2nd Weighing (g)*	% Change in Mass
**1**	0.9343	1.2826	20.4586	20.4585	0.0005
**2**	0.95	1.2766	28.1955	28.4213	0.0025
**3**	0.9011	1.2274	28.4925	28.4923	0.0007

*Mass (g) = Mass of crucible + Mass of filter paper + Mass of sample

Table S.II.4. Mass of cellulose content.

Trials	Mass of cellulose & filter paper (g)	Mass of filter paper (g)	Mass of cellulose (g)	% Cellulose
**1**	2.1875	1.2826	0.9049	90.49
**2**	2.1955	1.2766	0.9189	91.89
**3**	2.0999	1.2274	0.8725	87.25

Table S.II.5. Mass of hemicellulose & lignin and other extractives.

Trials	Mass of sample (g)	Mass of holocellulose (g)	Mass of cellulose (g)	Mass of hemicellulose (g)	Mass of lignin and other extractives (g)
**1**	1.0034	0.9343	0.9049	0.0294	0.0691
**2**	1.0025	0.95	0.9189	0.0311	0.0525
**3**	1.0084	0.9011	0.8725	0.0297	0.1073
**Average**		0.9284	0.8988	0.0297	0.0763

Supplementary III: Raw data and analysis via Response Surface Methodology and two-factor ANOVA of nanocellulose yield from maguey fiber

Table S.III.1. Mass of resulting post-hydrolysis cellulose from maguey fibers at temperatures 30, 40, 50, and 60°C and acid concentrations 40, 50, and 60% H2SO4.

		TRIAL 1			TRIAL 2			TRIAL 3		
**Temperature (°C)**	**Sulfuric Acid Concentration (% H_2SO_4)**	Mass of Post-hydrolysis Cellulose + Container (g)	Mass of Container (g)	**Mass of Post-hydrolysis Cellulose (g)**	Mass of Post-hydrolysis Cellulose + Container (g)	Mass of Container (g)	**Mass of Post-hydrolysis Cellulose (g)**	Mass of Post-hydrolysis Cellulose + Container (g)	Mass of Container (g)	**Mass of Post-hydrolysis Cellulose (g)**
**30**	**40**	6.8518	6.6810	**0.1708**	6.7596	6.5800	**0.1796**	6.8127	6.6264	**0.1863**
	**50**	7.0284	6.6923	**0.3361**	7.1654	6.8463	**0.3191**	6.9794	6.6747	**0.3047**
	**60**	7.9910	7.7792	**0.2118**	8.0109	7.7759	**0.2350**	7.9293	7.7052	**0.2241**
**40**	**40**	7.9840	7.7795	**0.2045**	7.9569	7.7386	**0.2183**	7.1009	6.8902	**0.2107**
	**50**	8.0613	7.7548	**0.3065**	8.0299	7.7440	**0.2859**	8.0529	7.7583	**0.2946**
	**60**	7.9810	7.7710	**0.2100**	7.9176	7.7174	**0.2002**	7.9945	7.7833	**0.2112**
**50**	**40**	8.1301	7.7440	**0.3861**	8.1221	7.7374	**0.3847**	8.1399	7.7641	**0.3758**
	**50**	8.1715	7.7555	**0.4160**	8.1893	7.7797	**0.4096**	8.1606	7.7444	**0.4162**
	**60**	7.0007	6.7369	**0.2638**	6.8486	6.5809	**0.2677**	6.9316	6.6541	**0.2775**
**60**	**40**	8.1088	7.7675	**0.3413**	8.1354	7.7859	**0.3495**	8.116	7.7749	**0.3411**
	**50**	8.1449	7.7813	**0.3636**	8.0884	7.7096	**0.3788**	8.077	7.7057	**0.3713**
	**60**	7.9844	7.7587	**0.2257**	8.0106	7.7775	**0.2331**	8.0096	7.7602	**0.2494**

Table S.III.2. Summary of calculated nanocellulose yield after acid hydrolysis at temperatures 30, 40, 50, and 60oC and at acid concentrations 40, 50, and 60% H2SO4.

		TRIAL 1			TRIAL 2			TRIAL 3		
**Temperature (°C)**	**Sulfuric Acid Concentration (% H_2SO_4)**	Mass of Extracted Cellulose (g)	Mass of Post-hydrolysis Cellulose (g)	**Post-hydrolysis Cellulose Yield (%)**	Mass of Extracted Cellulose (g)	Mass of Post-hydrolysis Cellulose (g)	**Post-hydrolysis Cellulose Yield (%)**	Mass of Extracted Cellulose (g)	Mass of Post-hydrolysis Cellulose (g)	**Post-hydrolysis Cellulose Yield (%)**
**30**	**40**	0.5055	0.1708	**33.7883**	0.5002	0.1796	**35.9056**	0.5012	0.1863	**37.1708**
	**50**	0.5073	0.3361	**66.2527**	0.5025	0.3191	**63.5025**	0.5048	0.3047	**60.3605**
	**60**	0.5044	0.2118	**41.9905**	0.5035	0.2350	**46.6733**	0.5028	0.2241	**44.5704**
**40**	**40**	0.5009	0.2045	**40.8265**	0.5061	0.2183	**43.1338**	0.5050	0.2107	**41.7228**

	**50**	0.5071	0.3065	**60.4417**	0.5061	0.2859	**56.4908**	0.5037	0.2946	**58.4872**
	**60**	0.5036	0.2100	**41.6998**	0.5018	0.2002	**39.8964**	0.5027	0.2112	**42.0131**
**50**	**40**	0.5010	0.3861	**77.0659**	0.5036	0.3847	**76.3900**	0.5014	0.3758	**74.9501**
	**50**	0.5062	0.4160	**82.1810**	0.5028	0.4096	**81.4638**	0.5084	0.4162	**81.8647**
	**60**	0.5050	0.2638	**52.2376**	0.5092	0.2677	**52.5727**	0.5033	0.2775	**55.1361**
**60**	**40**	0.5011	0.3413	**68.1102**	0.5014	0.3495	**69.7048**	0.5064	0.3411	**67.3578**
	**50**	0.5093	0.3636	**71.3921**	0.5075	0.3788	**74.6404**	0.5004	0.3713	**74.2006**
	**60**	0.5036	0.2257	**44.8173**	0.5058	0.2331	**46.0854**	0.5034	0.2494	**49.5431**

Table S.III.3. Summary of calculated average nanocellulose yields and their corresponding standard deviations.

	**40% H_2SO_4**		**50% H_2SO_4**		**60% H_2SO_4**	
	Average Nanocellulose Yield (%)	Standard Deviation	Average Nanocellulose Yield (%)	Standard Deviation	Average Nanocellulose Yield (%)	Standard Deviation
**30°C**	35.6216	1.7090	63.3719	2.9483	44.4114	2.3454
**40°C**	41.8944	1.1632	58.4732	1.9755	41.2031	1.1424
**50°C**	76.1353	1.0806	81.8365	0.3594	53.3155	1.5856
**60°C**	68.3909	1.1984	73.4110	1.7622	46.8153	2.4460

A two-way ANOVA was performed to analyze the effect of temperature and acid concentration employed during acid hydrolysis on the nanocellulose yield, and the results are presented in Table S.III.4-5. This two-way ANOVA has the null hypothesis that there is no significant difference in resulting nanocellulose yield between the means of the factor, either temperature or acid concentration. It also assumes that there is no significant interaction effect between the two independent variables. Simple main effects analysis showed that temperature has a statistically significant effect on nanocellulose yield. Simple main effects analysis also showed that acid concentration has a statistically significant effect on nanocellulose yield.

Table S.III.4. Summary of resulting parameters from ANOVA two-factor analysis without replication

SUMMARY	***Count***	***Sum***	***Average***	***Variance***
**30°C**	3	143.40	47.80	201.14
**40°C**	3	141.57	47.19	95.60
**50°C**	3	211.29	70.43	227.78
**60°C**	3	188.62	62.87	199.67
**40% H_2SO_4**	4	222.04	55.51	390.75
**50% H_2SO_4**	4	277.09	69.27	108.81
**60% H_2SO_4**	4	185.75	46.44	26.32

Table S.III.5. Anova: two-factor without replication on the effect of temperature and acid concentration on nanocellulose yield.

Source of Variation	***SS***	***df***	***MS***	***F***	***P-value***	***F crit***
Temperature	1186.94	3	395.646	6.076	0.030	4.757
Acid Concentration	1057.70	2	528.848	8.122	0.020	5.143
Error	390.69	6	65.116			
Total	2635.326	11				

"SS" refers to the sum of squares due to the source, which measures the deviation of data points away from the mean value. A higher result of SS indicates a higher variability of the resulting nanocellulose yield from the mean nanocellulose yield obtained from that source. In comparison, a lower result of SS indicates a low variability from the mean. "df" refers to the degrees of freedom in the source, which refers to the number of logically independent variables that are free to vary in the data sample. "MS" refers to the mean sum of squares SS due to the source. "F" refers to F-statistic or F-value which is the value used in ANOVA to determine the ratio of explained variance to unexplained variance and determine the significance of the differences in the means of a data set. "F crit" refers to the critical F-value, which is a specific value being compared with F to conclude whether to reject the null hypothesis or not. Generally, if F in a test is larger than your F-crit, the null hypothesis is rejected. "P-value" refers to the probability that the results are obtained by chance and are compared to the significance level, alpha (α), which is the probability of making the error to reject a true hypothesis and is usually set to 0.05. If the P-value is less than the α, then the null hypothesis is rejected.

Since the P-value for the temperature is less than 0.05 [$P - value = 0.030 < 0.05 = \alpha$ (or $F = 6.076 > 4.757 = F\ crit$)], we reject the null hypothesis. Thus, at the 95% confidence level, we conclude there is a significant difference in the nanocellulose yields produced by the varying temperatures (30, 40, 50, and 60 °C) during acid hydrolysis for 45 minutes. On the other hand, Since the p-value for the acid concentration is less than 0.05 [$P - value = 0.020 < 0.05 = \alpha$ (or $F = 8.122 > 5.143 = F\ crit$)], we reject the null hypothesis. Thus, at a 95% confidence level, we conclude there is a significant difference in the nanocellulose yields produced by the varying acid concentrations (40, 50, and 60% H_2SO_4) during acid hydrolysis for 45 minutes.

Moreover, undergoing ANOVA for a Multilevel Categoric Full Factorial Design Type via Response Surface Methodology of DX-Expert Version 22.0.3 Software, the analysis summarized as shown in the following tables.

Table S.III.6. ANOVA for selected factorial model.

Source of Variation	*SS*	*df*	*MS*	*F*	*P-value*	
**Model**	7906.00	11	718.73	226.90	<0.0001	Significant
A-Acid Concentration	3173.09	2	1586.55	500.88	<0.0001	Significant
B-Temperature	3560.81	3	1186.94	374.72	<0.0001	Significant
AB	1172.09	6	195.35	61.67	<0.0001	Significant
Pure Error	76.02	24	3.17			
Cor Total	7982.02	35				

The **Model F-value** of 226.90 implies the model is significant. There is only a 0.01% chance that an F-value this large could occur due to noise. **P-values** less than 0.0500 indicate model terms are significant. In this case A, B, AB are significant model terms.

Table S.III.7. Fit Statistics of the model.

Std. Dev.	*Mean*	*C.V. %*	*R^2*	*Adjusted R^2*	*Predicted R^2*	*Adeq. Precision*
1.78	57.07	3.12	0.9905	0.9861	0.9786	44.9761

The **Predicted R^2** of 0.9786 is in reasonable agreement with the **Adjusted R^2** of 0.9861; i.e. the difference is less than 0.2. **Adeq Precision** measures the signal to noise ratio. A ratio greater than 4 is desirable. The ratio of 44.976 indicates an adequate signal. This model can be used to navigate the design space.

From Design Expert, a final equation in terms of coded factors was obtained. The equation in terms of coded factors can be used to make predictions about the response for given levels of each factor. By default, the high levels of the factors are coded as +1 and the low levels are coded as -1. The coded equation is useful for identifying the relative impact of the factors by comparing the factor coefficients.

$$\text{Nanocellulose Yield} = 57.07 - 1.56\text{A} + 12.20\text{A}^2 - 9.27B - 9.88B^2 + 13.36B^3 - 10.62AB + 3.37A^2B - 3.73AB^2 - 0.9168A^2B^2 + 7.27AB^3 - 0.7924A^2B^3$$

where A refers to the acid concentration and Bis the reaction temperature.

The model graph for the acid concentration, temperature, and combined interactions are presented in the following figures.

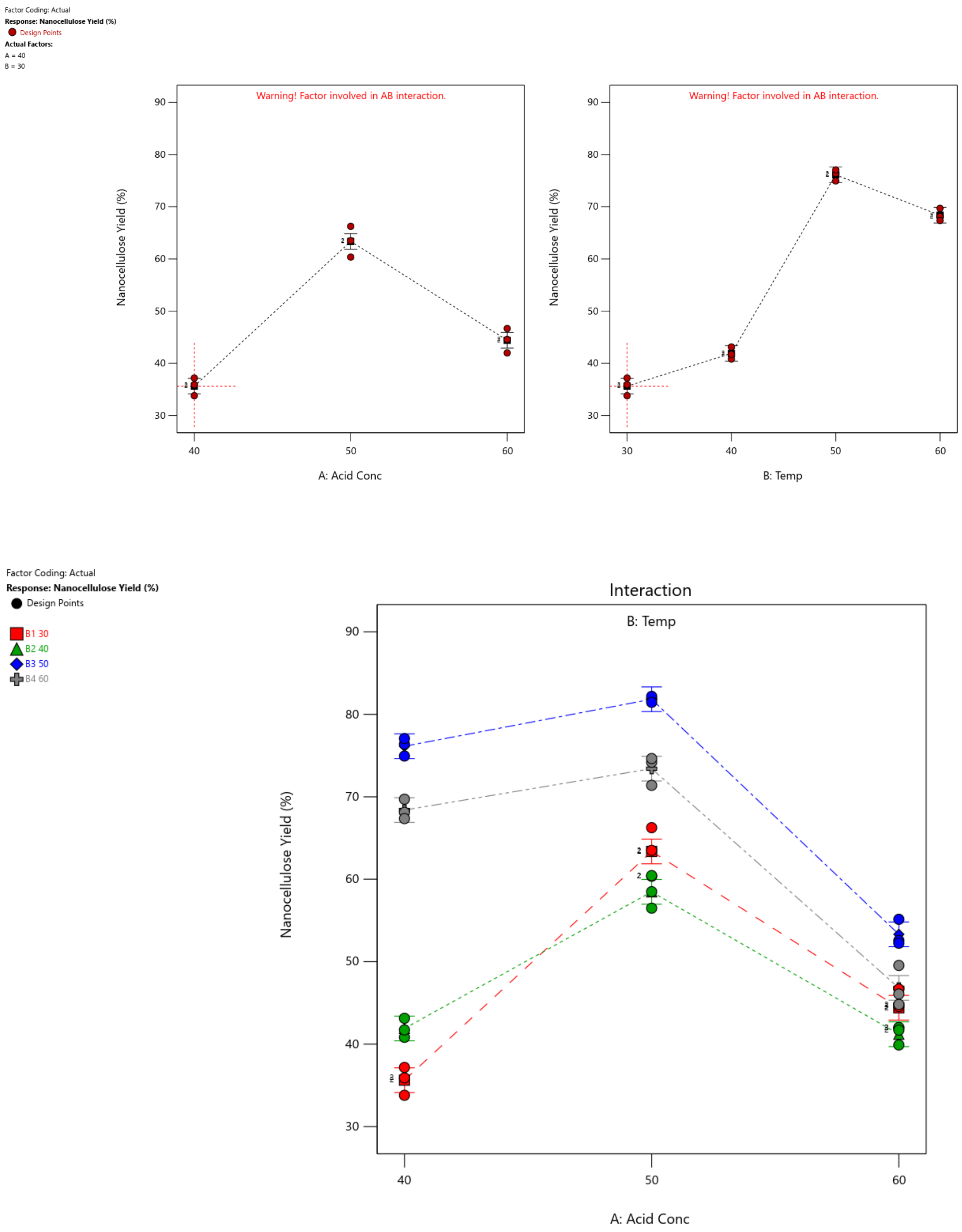

Figure S.III.1. DX-Expert model graphs.

Supplementary IV: Characterization of nanocellulose

S.IV.1. *<u>Morphological analysis of acid-hydrolyzed cellulose from maguey fiber</u>*

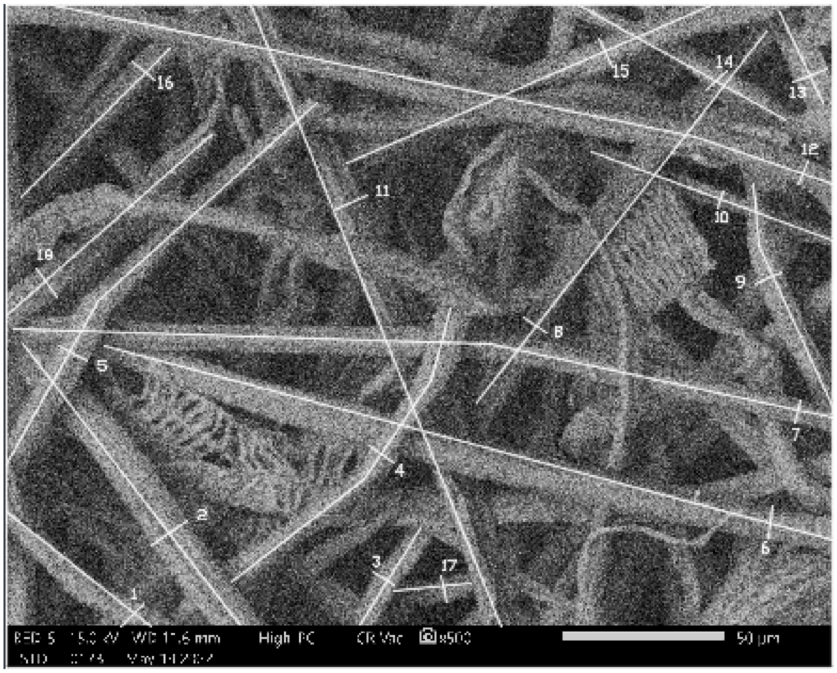

Table S. IV. 1. Cellulose Dimensions

No.	Relative Length (µm)	Relative Diameter (µm)	Length (µm)	Diameter (µm)
1	12.4977	2.1035	57.33416	9.649968
2	23.9782	2.9359	110.0018	13.46867
3	7.656	1.6451	35.12249	7.547023
4	24.4059	2.0046	111.9639	9.196257
5	32.5518	2.1906	149.3339	10.04955
6	50.8936	2.1768	233.4783	9.986237
7	55.8109	1.6256	256.0368	7.457565
8	31.7868	1.7728	145.8244	8.132856
9	16.0917	1.8547	73.82191	8.508579
10	17.301	1.1712	79.36967	5.37297
11	45.153	2.4668	207.1429	11.31663
12	54.1487	1.7315	248.4113	7.943389
13	6.7798	2.444	31.10285	11.21204
14	15.938	1.8731	73.1168	8.59299
15	26.8356	1.9961	123.1104	9.157262
16	14.1303	1.954	64.82384	8.964125
17	5.2088	1.7107	23.89577	7.847968
18	18.5088	2.319	84.91054	10.63859

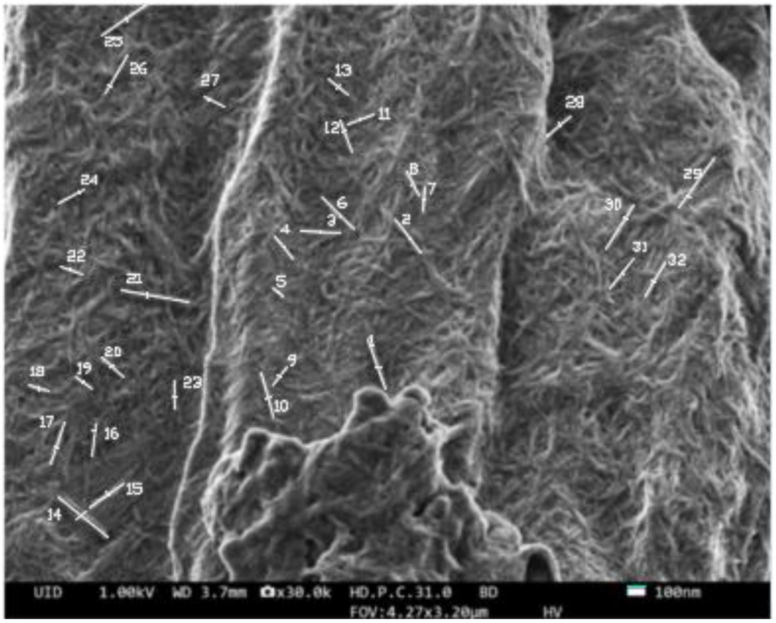

Table S. IV. 2. Nanocellulose dimensions from non-sonicated sample.

No.	Relative Length (nm)	Relative Diameter (nm)	Length (nm)	Diameter (nm)
1	1.4776	0.1518	288.3685	29.62529
2	1.1813	0.0521	230.5425	10.16784
3	1.1417	0.1008	222.8142	19.67213
4	0.7853	0.042	153.2592	8.196721
5	0.3713	0.046	72.46292	8.977361
6	1.2533	0.0782	244.5941	15.26151
7	0.6678	0.0632	130.3279	12.33411
8	0.7443	0.0715	145.2576	13.95394
9	0.6718	0.0992	131.1085	19.35988
10	1.2736	0.1346	248.5558	26.26854
11	0.722	0.093	140.9055	18.14988
12	0.9397	0.1049	183.3919	20.47229
13	0.6613	0.1132	129.0593	22.09212
14	1.7483	0.3603	341.1983	70.31616
15	1.2068	0.1201	235.5191	23.43872
16	0.894	0.1331	174.4731	25.9758
17	1.2284	0.1675	239.7346	32.68931
18	0.6075	0.0899	118.5597	17.54489
19	0.5648	0.0767	110.2264	14.96877
20	0.834	0.1342	162.7635	26.19048
21	1.9186	0.1433	374.434	27.96643
22	0.617	0.0739	120.4137	14.42233
23	0.7786	0.1163	151.9516	22.69711

24	0.8107	0.0692	158.2162	13.50507
25	1.5426	0.1169	301.0539	22.81421
26	3.1191	0.2699	608.7237	52.67369
27	1.5857	0.1519	309.4653	29.64481
28	2.1924	0.3839	427.8689	74.92194
29	4.4375	0.3345	866.0226	65.28103
30	3.6685	0.3127	715.9446	61.02654
31	2.7556	0.1908	537.783	37.23653
32	2.805	0.3811	547.4239	74.37549

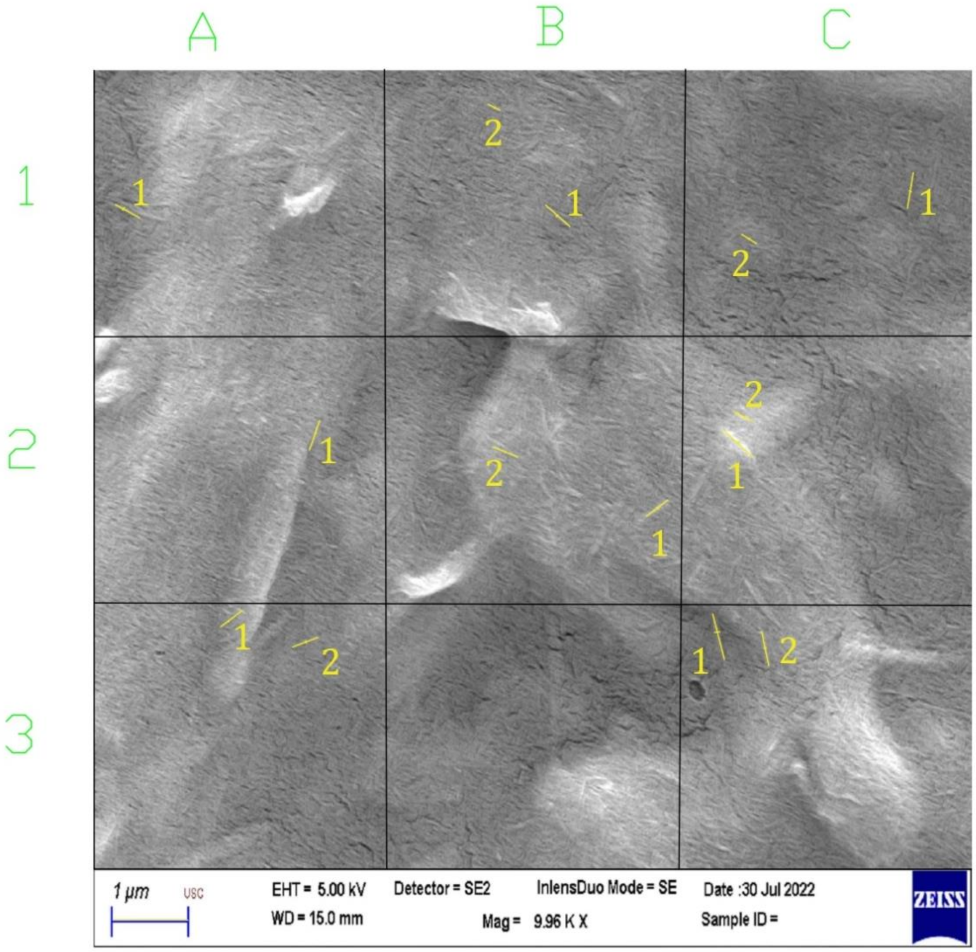

Table S. IV. 3. Nanocellulose dimensions from sonicated sample.

Quadrant	No.	Relative Length	Relative Diameter	Actual Length (nm)	Actual Diameter (nm)
A1	1	16.4311	1.7804	364.0119719	39.44269798
A2	1	18.7393	1.1449	415.147467	25.36393222
A3	1	7.9067	0.673	175.1637723	14.90953479

	2	16.3314	1.5405	361.803234	34.1279916
B1	1	19.4732	1.6062	431.4061707	35.58349893
	2	7.9067	0.673	175.1637723	14.90953479
B2	1	16.0392	1.703	355.3298818	37.72799071
	2	16.1773	0.9065	358.3893272	20.0824566
C1	1	21.6744	1.3687	480.1712049	30.32196177
	2	10.4921	1.0617	232.440312	23.52073267
C2	1	23.877	2.2622	528.9672544	50.11641843
	2	12.5307	1.1532	277.6031317	25.5478091
C3	1	28.1626	3.186	623.9097541	70.58213647
	2	21.1841	1.3517	469.3091768	29.94534647

S.IV.2. <u>*Transmission Electron Microscope (TEM) analysis*</u>

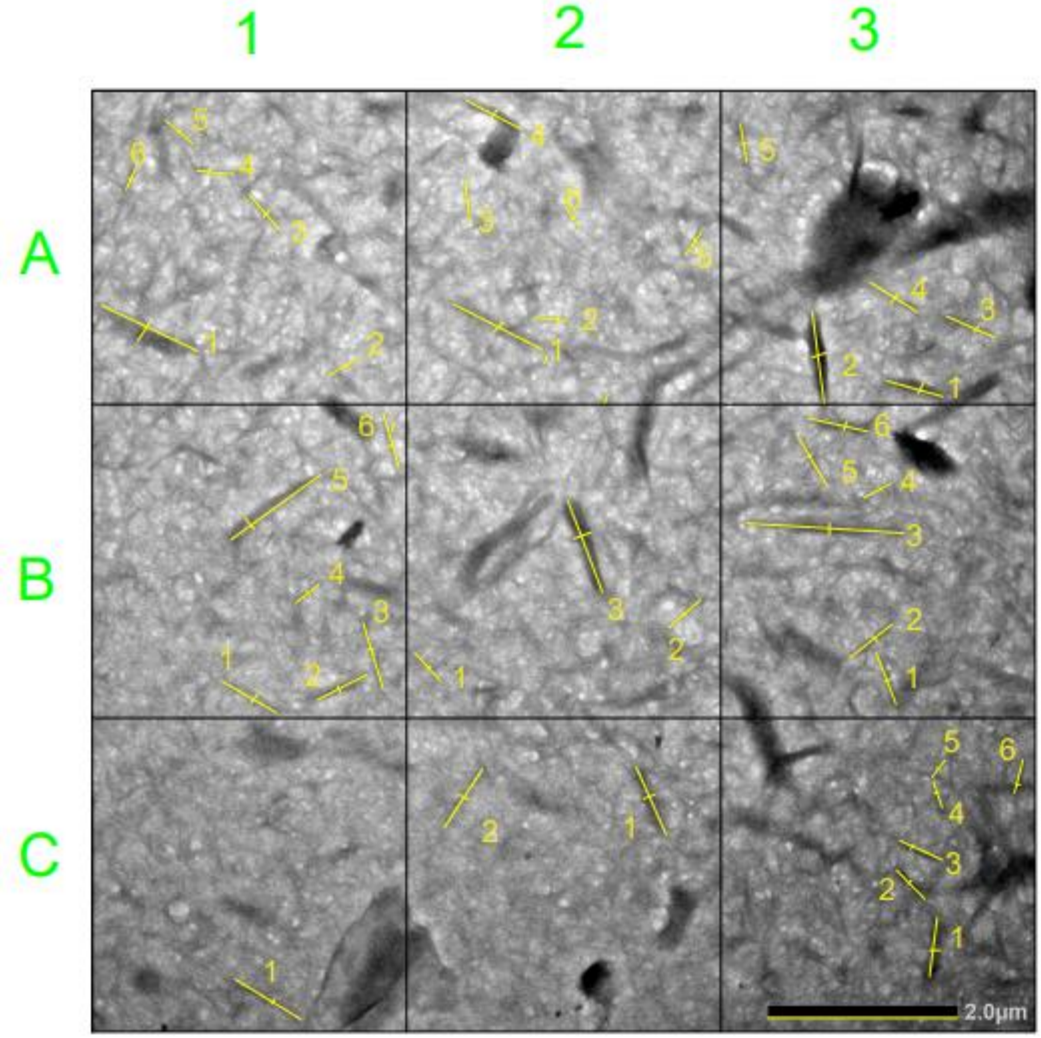

Quadrant	No.	Relative Length	Relative Diameter	Actual Length (nm)	Actual Diameter (nm)
A1	1	4.285	0.2962	385.1702704	26.62483876
	2	5.3954	0.9643	484.981955	86.67904125
	3	4.3938	0.8455	394.9500897	76.00034157
	4	4.2243	0.3396	379.7140661	30.52597989
	5	2.7747	0.5597	249.4123569	50.31033847
A2	1	3.7021	0.5952	332.7745293	53.50136405
	2	5.1114	0.6297	459.4537504	56.60250158
	3	7.3646	1.1176	661.9894921	100.4588785
	4	3.2176	0.729	289.2237718	65.5283844
	5	2.5855	0.519	232.4055389	46.65189506
A3	1	6.765	1.4051	608.0926206	126.3016912
	2	6.3601	0.8468	571.6969514	76.11719603
	3	6.523	1.329	586.3397139	119.4612111
	4	4.5152	0.888	405.8624983	79.82058346
B1	1	7.3551	1.2806	661.1355557	115.1106297
	2	6.4217	0.8793	577.234055	79.03855748
	3	8.2375	0.7466	740.4527661	67.11041398
	4	3.3747	0.5992	303.3451836	53.86091623
	5	6.5826	0.8357	591.6970413	75.11943874
B2	1	4.4209	0.4351	397.3860557	39.11028814
	2	4.8849	0.5142	439.0941083	46.22043245
B3	1	6.3877	0.7087	574.1778615	63.7036571
	2	6.3122	0.8726	567.3913141	78.43630758
	3	3.4775	0.4133	312.5856745	37.15072877
	4	6.7802	0.5714	609.4589189	51.36202859
	5	7.0543	1.0577	634.0972319	95.0745846
C1	1	9.0124	0.6946	810.1070117	62.43623567
C2	1	8.667	1.4118	779.0596812	126.9039411
	2	8.2064	1.2584	737.6572479	113.1151151
C3	1	6.9645	1.2459	626.0252855	111.9915146
	2	4.7069	0.5743	423.0940364	51.62270392
	3	5.6026	0.5857	503.6067578	52.64742763
	4	3.1866	0.4639	286.4372424	41.69906382
	5	2.5592	0.4578	230.0414833	41.15074674
	6	3.9915	0.5805	358.7881294	52.1800098

S.IV.3. ***<u>Fourier Transform Infrared (FTIR) Spectroscopy Analysis</u>***

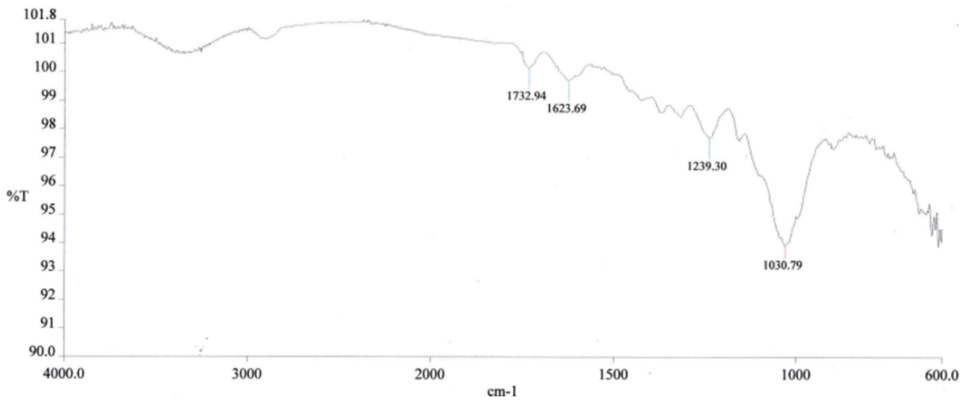

Figure S.IV.3.1. FTIR Spectrum for Raw Maguey Fibers

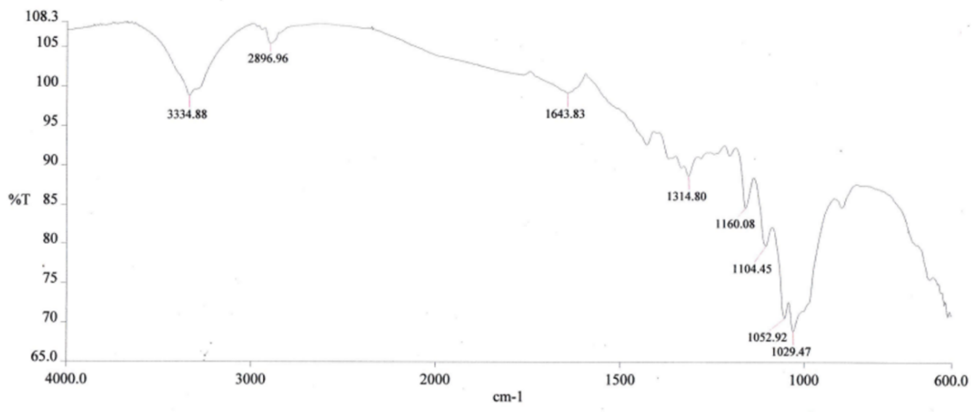

Figure S.IV.3.2. FTIR Spectrum for Cellulose

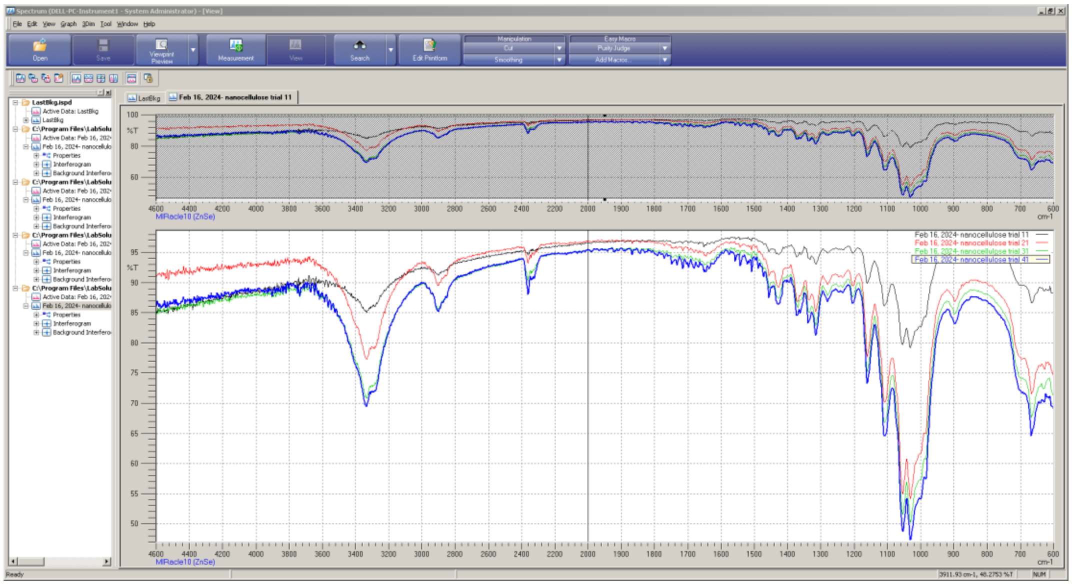

Figure S.IV.3.3. FTIR Spectrum for Nanocellulose

S.IV.4. *<u>Zeta Potential Analysis Results</u>*

2024.04.14 14:37:43

HORIBA Scientific

HORIBA SZ-100 for Windows [Z Type] Ver2.50

SZ-100

Measurement Results

20240414_Nanocellulose_4872.nzt

Measurement Results

Date	:	Sunday, April 14, 2024 2:28:34 PM
Measurement Type	:	Zeta Potential
Sample Name	:	20240414_Nanocellulose
Temperature of the Holder	:	25.0 °C
Dispersion Medium Viscosity	:	0.895 mPa·s
Conductivity	:	0.285 mS/cm
Electrode Voltage	:	3.3 V

Calculation Results

Peak No.	Zeta Potential	Electrophoretic Mobility
1	-37.3 mV	-0.000289 cm2/Vs
2	--- mV	--- cm2/Vs
3	--- mV	--- cm2/Vs

Zeta Potential (Mean)	:	-37.3 mV
Electrophoretic Mobility Mean	:	-0.000289 cm^2/Vs

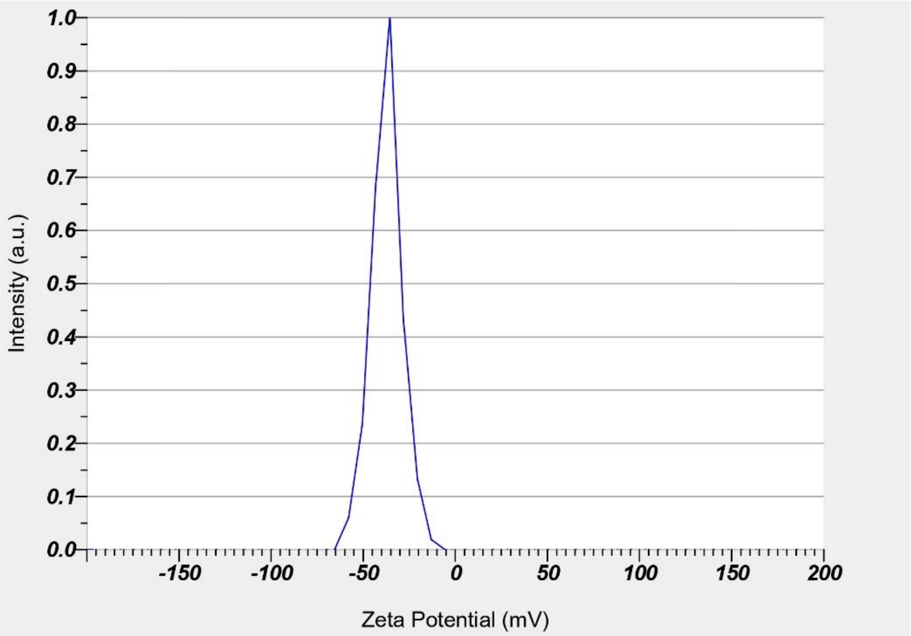

Explore the future | Automotive Test Systems | Process & Environmental | Medical | Semiconductor | Scientific | HORIBA

1 / 1

Figure S.IV.4.1. Zeta Potential Analysis Results

S.IV.5. *X-ray Diffraction (XRD) Analysis Profile Data*

Profile Data Ascii Dump (XRD)

Group : Standard
Data : LeysonNC
File Name : LeysonNC.RAW

```
# Profile Datafile
        comment          = LeysonNC
        date & time      = 07-27-22 09:12:58

# Measurement Condition
    X-ray tube
        target           = Cu
        voltage          = 40.0 (kV)
        current          = 30.0 (mA)
    Slits
        divergence slit  = 1.00000 (deg)
        scatter slit     = 1.00000 (deg)
        receiving slit   = 0.30000 (mm)
    Scanning
        drive axis       = Theta-2Theta
        scan range       =    2.000 -   70.000
        scan mode        = Continuous Scan
        scan speed       =   1.0000 (deg/min)
        sampling pitch   =   0.0200 (deg)
        preset time      =   1.20 (sec)
```

Republic of the Philippines
Department of Science and Technology
INDUSTRIAL TECHNOLOGY DEVELOPMENT INSTITUTE
ADVANCED DEVICE AND MATERIALS TESTING LABORATORY
DOST Cpd., General Santos Ave., Bicutan, Taguig City
Tel. Nos. (Direct Line): (02) 8837-0461, 8837-0503, 8837-0674, 8837-0650
http://www.itdi.gov.ph, http://www.admatel.com

PAB Accredited
Testing Laboratory
PNS ISO/IEC 17025:2017
LA-2015-271B

REPORT OF ANALYSIS

**Reference No.**	:	**ADMATEL 2207-3488**
**Customer**	:	**UNIVERSITY OF SAN CARLOS**<br>Andrea Kaylie B. Leyson<br>Department of Chemical Engineering<br>16100815@usc.edu.ph
**Sample Label**	:	Nanocellulose (in solid white powder form)
**Analysis Requested**	:	Thermogravimetric – Differential Thermal Analysis (TG-DTA)
**Date Received**	:	July 13, 2022
**Date Tested**	:	July 19, 2022

I. Test Description

Measuring Cell	:	Perkin Elmer STA 6000
Sample Holder	:	Ceramic Crucible
Temperature Program	:	Heating from 30˚C to 600˚C with heating rate of 10˚C/min
Atmosphere	:	Nitrogen at 20 mL/min from 30˚C to 600˚C

II. Summary

The photograph, Thermogravimetric – Differential Thermogravimetric (TG-DTG) and Differential Thermal Analysis (DTA) curves of the sample are shown in **Figures 1** and **2**. Evident weight losses and corresponding peak temperatures in the TG-DTG curves and DTA peak temperatures are presented in **Tables 1** and **2**, respectively. The obtained weight of the sample is presented in **Table 3**.

This report shall not be reproduced in any form, except in full, without written approval of ADMATEL

III. Results

Table S.IV.6.1. TG-DTG Data.

Sample ID	Weight Loss, % with corresponding peak temperature	Peak Temperature[b] (T_P), °C	Temperature Range, °C
Nanocellulose (in solid white powder form)	Weight loss 1 = 4.715<br>Weight loss 2 = 81.223<br>Residue[a] = 14.062<br>Total = 100.000	59.31<br>311.41	30.00 – 129.13<br>129.13 – 427.81

Remarks:
[a] Refers to residual weight obtained at the final temperature.
[b] The first derivative peak temperature indicated the point of greatest rate of change on the weight loss curve, also known as the inflection point.

Table S.IV.6.2. DTA peak temperatures.

Sample ID	Endothermic Peak Temperatures (T_P), °C
Nanocellulose (in solid white powder form)	T_{p1} = 66.39<br>T_{p2} = 307.47

IV. Remarks

The following weight shown in **Table 3** was obtained from the submitted sample and analyzed using the Simultaneous Thermal Analyzer (STA).

Table S.IV.6.3. Obtained weight of the sample.

Sample ID	Weight, mg
Nanocellulose (in solid white powder form)	13.549

This report shall not be reproduced in any form, except in full, without written approval of ADMATEL

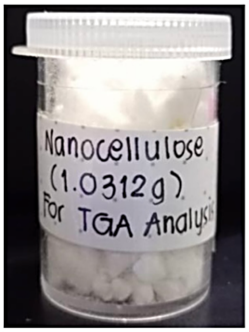

Figure S.IV.6.1. Photograph of as-received *Nanocellulose (in solid white powder form)* sample.

VALIDITY OF THE REPORT: The test results are those obtained at the time of the test and pertain only to the sample/s received by ADMATEL.

LYNNE JERISA A. CASTRO
Laboratory Analyst
Date: 07/21/2022

ANGELENE J. ALCAIN
Laboratory Head
Date: 07/21/2022

Issued under the authority of:

ARACELI M. MONSADA, Dr. -Eng'g.
Laboratory Manager
Date: 07/21/2022

Form: AL-21-F25c
Issue: December 14, 2020
Revision: 02

This report shall not be reproduced in any form, except in full, without written approval of ADMATEL

Filename:	c:\program ...\nanocellulose processed.stad
Operator ID:	Gabunada
Sample ID:	Nanocellulose
Sample Weight:	13.549 mg
Comment:	2207-3488 University of San Carlos

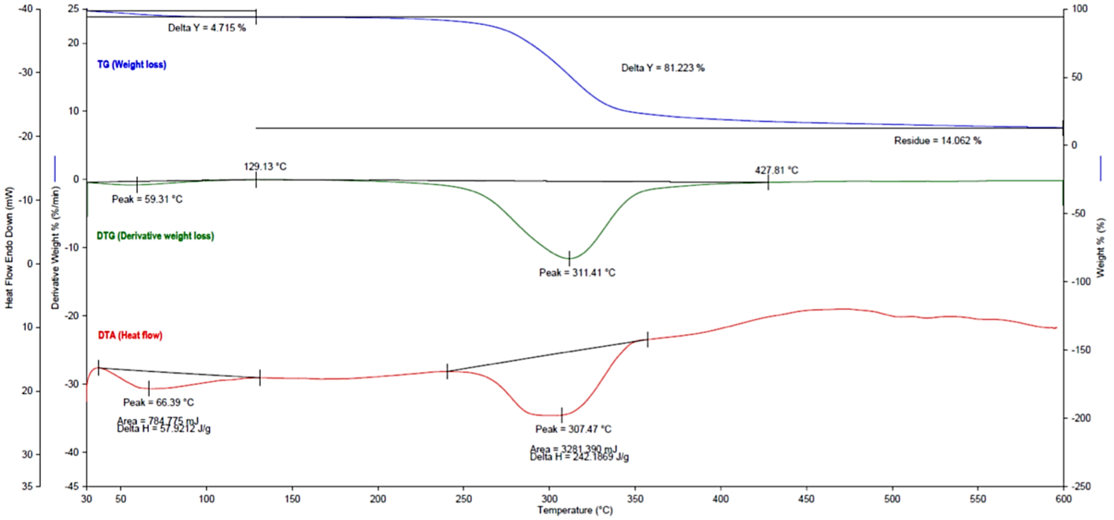

Figure S.IV.6.2. TG-DTG-DTA curve of the *Nanocellulose (in solid white powder form)* sample.

This report shall not be reproduced in any form, except in full, without written approval of ADMATEL
